# The Epithelial–Mesenchymal Transcription Factor *SNAI1* Represses Transcription of the Tumor Suppressor miRNA *let-7* in Cancer

**DOI:** 10.3390/cancers13061469

**Published:** 2021-03-23

**Authors:** Hanmin Wang, Evgeny Chirshev, Nozomi Hojo, Tise Suzuki, Antonella Bertucci, Michael Pierce, Christopher Perry, Ruining Wang, Jeffrey Zink, Carlotta A. Glackin, Yevgeniya J. Ioffe, Juli J. Unternaehrer

**Affiliations:** 1 Division of Biochemistry, Department of Basic Sciences, Loma Linda University, Loma Linda, CA 92354, USA; hwang1@students.llu.edu (H.W.); echirshev@students.llu.edu (E.C.); nozomi.hojo@riken.jp (N.H.); tsuzuki@students.llu.edu (T.S.); abertucci@llu.edu (A.B.); cperry@llu.edu (C.P.); 2 Department of Biology, California State University San Bernardino, San Bernardino, CA 92407, USA; michaelpierce@llu.edu; 3 Department of Chemistry and Biochemistry, University of California, Los Angeles, CA 90095, USA; wangrn8600@gmail.com (R.W.); zink@chem.ucla.edu (J.Z.); 4 Beckman Research Institute, City of Hope, Duarte, CA 91016, USA; CGlackin@coh.org; 5 Division of Gynecologic Oncology, Department of Obstetrics and Gynecology, Loma Linda University Medical Center, Loma Linda, CA 92354, USA; YIoffe@llu.edu; 6 Center for Health Disparities and Molecular Medicine, Loma Linda University, Loma Linda, CA 92354, USA

**Keywords:** epithelial–mesenchymal transition, stem cells, ovarian cancer, transcriptional regulation, miRNA, orthotopic patient-derived xenografts

## Abstract

**Simple Summary:**

When cells undergo epithelial–mesenchymal transition (EMT) they gain characteristics of stem cells. We investigated the mechanism by which the EMT transcription factor *SNAI1* induces stem cell features. In these studies, we observed that *SNAI1* represses a microRNA that maintains differentiation, *let-7*. This microRNA is lost in cancer, and its loss correlates with poor prognosis. In breast, pancreatic, and ovarian cancer cell lines the cell stemness in increased by *SNAI1* overexpression and reduced by *SNAI1* knockdown. We extended the ovarian cancer results to patient-derived cells, and to a mouse xenograft model. In mice, we used nanoparticles to deliver small RNAs (RNAi) targeting *SNAI1*, resulting in restoration of *let-7* levels, inhibition of stemness, and reduced tumor burden. Our studies validate nanoparticle-delivered RNAi targeting *SNAI1* as a clinically relevant approach.

**Abstract:**

We aimed to determine the mechanism of epithelial–mesenchymal transition (EMT)-induced stemness in cancer cells. Cancer relapse and metastasis are caused by rare stem-like cells within tumors. Studies of stem cell reprogramming have linked *let-7* repression and acquisition of stemness with the EMT factor, *SNAI1*. The mechanisms for the loss of *let-7* in cancer cells are incompletely understood. In four carcinoma cell lines from breast cancer, pancreatic cancer, and ovarian cancer and in ovarian cancer patient-derived cells, we analyzed stem cell phenotype and tumor growth via mRNA, miRNA, and protein expression, spheroid formation, and growth in patient-derived xenografts. We show that treatment with EMT-promoting growth factors or *SNAI1* overexpression increased stemness and reduced *let-7* expression, while *SNAI1* knockdown reduced stemness and restored *let-7* expression. Rescue experiments demonstrate that the pro-stemness effects of *SNAI1* are mediated via *let-7*. In vivo, nanoparticle-delivered siRNA successfully knocked down *SNAI1* in orthotopic patient-derived xenografts, accompanied by reduced stemness and increased *let-7* expression, and reduced tumor burden. Chromatin immunoprecipitation demonstrated that *SNAI1* binds the promoters of various *let-*7 family members, and luciferase assays revealed that *SNAI1* represses *let-7* transcription. In conclusion, the *SNAI1*/*let-7* axis is an important component of stemness pathways in cancer cells, and this study provides a rationale for future work examining this axis as a potential target for cancer stem cell-specific therapies.

## 1. Introduction

Cancer stem-like cells (CSC) are the subpopulation of tumor cells responsible for long-term maintenance of tumors. These cells are capable of self-renewal and differentiation, making them an important contributor to tumor recurrence [1]. The origin of CSC is not completely understood. In some cancers, normal tissue stem cells appear to be altered to result in CSC [1,2,3,4], while in others, somatic cells appear to be reprogrammed to the stem cell fate [5,6,7]. Whether the cells of origin in carcinomas are tissue resident stem cells or reprogrammed somatic cells, some aspects of the process by which CSC attain stem cell features are comparable to somatic cell reprogramming [4,7,8,9]. In somatic cell reprogramming, cells lose their differentiated characteristics and take on an embryonic or stem cell phenotype. Similarly, stem cells in tumors dedifferentiate and express genes consistent with the oncofetal state [10,11,12].

An important factor in maintenance of the differentiated state is the tumor suppressor miRNA *let-7*. *Let-7*, consisting in humans of nine highly conserved members in eight chromosomal locations, plays crucial roles in differentiation [13]. Because the individual family members’ seed sequence is identical, and the remaining sequence is different at only 1–3 residues, this miRNA family is generally presented as having redundant roles [13]. In pluripotent cells and germ cells, miRNA *let-7* expression is low, while differentiated cells uniformly express high levels [14]. Factors required for stemness (a property referring to a cell’s ability to self-renew and differentiate [3]) are inhibited by *let-7* [15]. Loss of *let-7* is thus necessary for the stem cell state, either in reprogramming or in cancer [13,16,17]. *Let-7* represses a set of embryonic genes and oncogenes, and its loss allows upregulation of those genes, resulting in the oncofetal state [13,14,15,16]. Replacing *let-7* reduces the stem cell population and reduces resistance to chemotherapy [18]. These data strongly implicate *let-7* as a key regulator of the CSC phenotype.

*Let-7* is frequently reduced in many types of cancer [13]. *Let-7* loss correlates with poor prognosis, functions as a biomarker for less differentiated cancer [13,19,20,21], and predicts tumor growth and metastasis [22]. Mechanisms for its loss are incompletely understood. miRNAs are regulated transcriptionally, epigenetically, and post-transcriptionally [23]. The pluripotency-associated factor *LIN28* blocks *let-7* biogenesis by inhibiting its processing to the mature form, but *LIN28* is downregulated in differentiated cells [20]. In addition to the post-transcriptional regulation of *let-7* by Lin28, transcriptional regulation of this miRNA family is an important factor in determining overall levels [23]. Several factors have been shown to regulate *let-7* transcription, including the epithelial–mesenchymal transition (EMT) transcription factor TWIST1, TP53, MYC, BMI1, NFKB1, and CEBPA [18]. We set out to study *let-7* regulation at the transcriptional level, because of evidence for its importance in dedifferentiation [17] and potential influence on the metastatic disease course.

EMT is a fundamental process for development and homeostasis whereby epithelial cells lose their cell polarity and cell–cell adhesion, and gain the migratory and invasive features typical of mesenchymal cells [21]. The aberrant activation of EMT is considered to be a hallmark of cancer metastasis [21,24,25]. Many studies have found that EMT is not an all-or-none response; instead, it is a multi-step process, with cells existing in states ranging from fully epithelial to fully mesenchymal. Cells are observed in several intermediate or partial (hybrid) EMT states [25]. In fact, cancer cells that undergo partial EMT (cells without complete loss of epithelial morphology or complete acquisition of mesenchymal morphology) have been reported to pose a higher metastatic risk [26,27]. Besides metastasis, cancer cells that undergo EMT demonstrate enhanced stemness, including tumor initiation ability and capacity to differentiate to multiple lineages [2,28,29]. The subpopulation within cancer cells with higher stemness has been shown to contribute to the tumor’s invasiveness and resistance to therapies [30,31]. A theoretical framework has linked EMT to stemness via cross-regulation of *let-7,* LIN28, and the miRNA-200 family [32]. However, few specific mechanistic studies show the molecular connections between EMT transcription factors and loss of the differentiated state. Yang et al. reported direct repression of *let-7i* by TWIST1 [33], leading us to ask whether another EMT transcription factor, SNAI1, plays a similar role. Hence, targeting stem-like cancer cells via EMT may be a crucial step to improve patient outcome.

Much evidence connects EMT with the acquisition of stem cell properties. Cells that have undergone EMT acquire the ability to differentiate to multiple lineages [28]. The expression of EMT transcription factors *SNAI1*, *SNAI2*, *TWIST1*, or *ZEB1* results in an increase in the proportion of cells with stem cell properties [29,34,35,36]. Recent work demonstrates that *SNAI1* expression locks cells in the hybrid state [37], and there is a computational report of *SNAI2* stabilizing the hybrid state as well [38]. Hence, cells with hybrid EMT properties have high likelihood of gaining stemness [32,37]. *SNAI1′*s transcription factor roles include repression of epithelial factors such as *CDH1*, stimulation of mesenchymal factors, and repression of miRNAs such as miR-34 [21,39]. We chose to focus on the EMT factor *SNAI1* because of its role in reprogramming somatic cells to pluripotency [17,40] and in cancer stemness [29,35,41]. 

Furthermore, *SNAI1* interacts with the miRNA of interest—*let-*7. It binds *let-7* family promoters and its early upregulation in reprogramming correlates with loss of *let-7* [17]. Because the increase of *SNAI1* and the decrease of *let-7* occurred at time points in reprogramming prior to upregulation of *LIN28*, we hypothesized that it might be the loss of *let-7*, rather than the gain of *LIN28*, that destabilized the differentiated state. In the studies presented here, we asked whether these reprogramming principles applied in cancer: Does expression of *SNAI1* lead to loss of *let-7* and gain of stemness?

A promising approach targeting genes, such as EMT transcription factors, includes a suitable antisense oligonucleotide strategy. However, technical challenges for such techniques include avoiding degradation by ubiquitous nucleases, preventing immune activation, and allowing extravasation and cellular uptake by targeted cells [42]. Poor cytoplasmic delivery of RNA therapeutics to appropriate cells has inhibited research progress, but our team has optimized a targeted nanoparticle delivery method to deliver RNAis to tumors [43]. Mesoporous silica nanoparticles (MSN) are small (50–200 nm), but have relatively large surface area due to their pore structure [44]. Coating them with cationic polyethylenimine (PEI) facilitates loading of siRNA cargo, and conjugation with hyaluronic acid (HA) assists delivery to target cells [45,46]: HA is the ligand for CD44, enriched on the surface of ovarian cancer stem cells [47].

In this study, we hypothesized that *SNAI1* directly represses miRNA *let-7* transcription, and that *SNAI1* knockdown would result in restoration of *let-7* expression and reduction of stemness and tumor growth. Using breast, pancreatic, and ovarian cancer cells, transforming growth factor beta-1 (TGFB1) or epidermal growth factor (EGF) treatment or *SNAI1* overexpression increased stemness and reduced *let-7* expression, while *SNAI1* knockdown reduced stemness and increased *let-7* expression. We demonstrate on the molecular level that *SNAI1* binds promoters of *let-7* family members in cancer cells. Luciferase assays demonstrate that the presence of *SNAI1* reduces *let-7* transcription, consistent with direct repression of *let-7* by *SNAI1*. Thus, one mechanism by which EMT promotes stemness is via loss of *let-7*, destabilizing the differentiated state. With the utilization of the orthotopic patient-derived xenograft (PDX) murine models of high grade serous ovarian carcinoma (HGSOC), we demonstrate feasibility of in vivo *SNAI1* knockdown by delivering siRNA with mesoporous silica nanoparticles. In orthotopic PDX, *SNAI1* knockdown results in increased *let-7* levels and reduced tumor growth.

## 2. Materials and Methods

### 2.1. Cell Cultures

The human HGSOC cell line OVSAHO (RRID:CVCL_3114) was the kind gift of Gottfried Konecny (University of California, Los Angeles, CA, USA), and OVCAR8 (RRID:CVCL_1629) was from Carlotta Glackin (City of Hope, Duarte, CA, USA). HEK293T (RRID:CVCL_0063), PANC-1 (RRID:CVCL_0480) (gift of Nathan Wall, Loma Linda University (LLU), Loma Linda, CA USA), MCF-7 (RRID:CVCL_0031) (gift of Eileen Brantley, LLU), OVSAHO and OVCAR8 cells were cultured in Dulbecco’s Modification of Eagle’s Medium (DMEM, GenClone, San Diego, CA, USA) with 10% fetal bovine serum (FBS, Omega Scientific, Tarzana, CA, USA), 2 mM of L-Glutamine, 100 U/mL of penicillin, and 10 µg/mL of streptomycin. NCCIT (RRID:CVCL_1451), used as a positive control for expression of pluripotency factors, was cultured in RPMI with 10% FBS, 2 mM L-Glutamine, 1 mM sodium pyruvate, 100 U/mL of penicillin, and 10 µg/mL of streptomycin. MCF-7 and PANC-1 cells were treated with TGFB1 (10 ng/mL), OVCAR8 and OVSAHO cells were treated with EGF (100 ng/mL). PDX6, a HGSOC chemotherapy naïve sample, was obtained as described [22]. Deidentified fresh ovarian cancer ascites samples was provided by the LLU Biospecimen Laboratory and were processed by centrifuging. Erythrocytes were removed by overlaying a cell suspension on a 3 mL Ficoll gradient. Cells were initially engrafted into NSG mice subcutaneously in the region of the mammary fat pad, resulting in PDX. Patient-derived samples were cultured in three parts Ham’s F12 and one part DMEM, supplemented with 5% FBS, 10 µM insulin, 0.4 µM hydrocortisone, 2 µg/mL isoprenaline, 24 µg/mL adenine, 100 U/mL of penicillin, and 10 µg/mL streptomycin. 5–10 µM Y27632 was added to establish growth in vitro [48]. Low passage (maximal passage number: 15) patient-derived cells were used to avoid changes induced by extensive passaging in in vitro culture. All human cell lines have been authenticated using STR profiling within the last three years. All experiments were performed with mycoplasma-free cells.

### 2.2. Institutional Review Board (IRB) Statement:

All subjects gave their informed consent before participation in the study. All studies were approved by the Loma Linda University (LLU) IRB (#58238, approved 24 January 2018). Investigations were carried out following the rules of the Declaration of Helsinki of 1975.

### 2.3. Reverse-transcription Quantitative PCR (RT-qPCR)

Total RNA from cell culture samples was isolated using TRIzol reagent (Life Technologies, Carlsbad, CA, USA) according to the manufacturer’s instructions. For mRNA expression analysis, cDNA was synthesized with 1 µg of total RNA using Maxima First Strand cDNA Synthesis Kit (K1672; Thermo Fisher scientific, Grand Island, NY, USA). Real-time RT-qPCR for mRNA was performed using PowerUP SYBR Green master mix (Thermo Fisher scientific, Grand Island, NY, USA) and specific primers on a Stratagene Mx3005P instrument (Agilent Technology, Santa Clara, CA, USA). Primer sequences are listed in Table S2. For miRNA expression analysis, cDNA was synthesized with 100 ng of total RNA using specific stem-loop RT primers and TaqMan microRNA Reverse Transcription Kit (Applied Biosystems, Foster City, CA, USA). Real-time RT-qPCR for miRNA was performed using TaqMan Universal PCR Master Mix II (Applied Biosystems, Foster City, CA, USA) with specific probes (Life Technologies 4440887 assay numbers 000377 (let-a), 002406 (let-7e), 002282 (let-7g), 002221 (let-7i), U47 (001223)) on a Stratagene Mx3005P instrument (Agilent Technology, Santa Clara, CA, USA). The results were analyzed using the cycles to threshold (Ct) method; ACTB (mRNA) and U47 (miRNA) were used for normalization.

### 2.4. Western Blot

Proteins were extracted from cells in PBS by adding SDS sample buffer (2% SDS, 2.5% beta-mercaptoethanol, 7.5% glycerol) and then sonicated for 10–15 s. Thirty microliters of lysate per sample (2.4 × 10^5^ cells) were heated to 100 °C for 5 min and then loaded on SDS-PAGE gel (4–12%). After running at 150 V for 20–40 min, samples were transferred to PVDF membrane. Membranes were incubated in 5% milk for blocking for 1 h at room temperature. After blocking and washing with 1X TBST, membranes were incubated in primary antibodies diluted at the appropriate dilution (as suggested by manufacturer data sheets) over night at 4 °C. Antibodies used include: HMGA2 (D1A7, Cell Signaling Technology, Danvers, MA, USA), SNAI1 (L70G2; Cell Signaling Technology, Danvers, MA, USA), α/β-TUBULIN (2148S; Cell Signaling Technology, Danvers, MA, USA), LIN28A (A177; Cell Signaling Technology, Danvers, MA, USA). Secondary antibody incubations were done with an anti-mouse IgG conjugated with DyLight 800 (SA5-10176; Invitrogen, Carlsbad, CA, USA) or anti-rabbit IgG antibody conjugated with DyLight 680 (35569; Invitrogen, Carlsbad, CA, USA) at 1/30,000 for 1 h at room temperature. Immunoblots were scanned and visualized using Odyssey Infrared Imaging System (LI-COR Biosciences, Lincoln, NE, USA). Densitometry was performed on scanned immunoblots by ImageJ software (National Institutes of Health, Bethesda, MD, USA). Quantification of Western blot data was done by measuring the intensity of bands of the protein of interest divided by the intensity of the samples’ own α/β-TUBULIN bands (ImageJ). All uncropped Western blot figures can be found in Figure S12.

### 2.5. Retroviral Overexpression

The cDNA of human *SNAI1* was subcloned from Flag-Snail WT (Addgene 16218, Watertown, MA, USA) into pWZL-Blast-GFP (Addgene 12269) after removing GFP using BamH1/Xho1. Retroviral particles were produced in HEK293T cells after co-transfection of retrovirus plasmid vector pWZL-Blast-Flag-Snail or control vector pWZL-Blast-Flag-Empty with packaging plasmids (VSVG, Gag/pol) using polyethylenimine (PEI) (Polysciences, Warrington, PA, USA). After 48 h and 72 h, supernatant containing virus was collected and filtered through a 0.22 μM filter. Supernatants were used for cell transduction or stored at −80 °C. Cells were transduced with retrovirus in the presence of 6 μg/mL protamine sulfate and selected with 5 µg/mL Blasticidin (InvivoGen #ant-bl-05 San Diego, CA, USA) for 5 days.

### 2.6. DsiRNA-Mediated Knockdown

A panel of dicer-substrate small inhibitory RNAs (DsiRNA, Integrated DNA Technologies (IDT), Coralville, IA, USA) were screened for SNAI1 knockdown (Figure S3). HA-conjugated, PEI-coated MSNs were synthesized as described [45]. Briefly, MSNs were produced using the sol-gel method, dissolving 250 mg cetyltrimethylammonium bromide in 120 mL water with 875 µL of 2 M sodium hydroxide solution. Next, 1.2 mL tetraethylorthosilicate was added, stirred for 2 h, allowing formation of MSN. Particles were collected by centrifugation and washed with methanol and acidic methanol. Low molecular weight cationic PEI (1.8 kDa branched polymer) was electrostatically attached to the MSN surface to provide a positive charge to attract negatively charged siRNA [45], and HA was covalently bound to the amine groups in the PEI using EDC-NHS coupling reaction [49]. DsiRNA targeting SNAI1 or control (oligonucleotide sequence listed in Table S4) were used for knockdown in vitro, loaded on MSN as described [43]. To complex siRNA for in vitro experiments, 10 μL siRNA at 10 μM was mixed with 70 μL MSNs at 500 μg/mL and 20 μL water, and the mixture was incubated overnight at 4 °C on a rotor. The following day, 100 μL of the HA-MSN-siRNA complexes were added to each well of a 6-well plate containing 1900 μL normal medium. To complex siRNA for in vivo experiments, 15 μL siRNA at 10 μM was mixed with 105 μL HA-MSNs at 500 μg/mL, and the mixture was incubated overnight at 4 °C on a rotor. The following day, 120 μL of the HA-MSN-siRNA complexes were injected intravenously (tail vein). For in vivo experiments, HA-MSN-siRNA were injected twice weekly.

### 2.7. Mimic Transfection

Let-7i mimics (sense: 5′-mCmArGmCrAmCrAmArAmCrUmArCmUrAmCrCmUrCA-3′; antisense 5′-/5Phos/rUrGrArGrGrUrArGrUrArGrUrUrUrGrUrGrCrUmGmUrU-3′) and scrambled control mimics (sense 5′-mCmArUmArUmUrGmCrGmCrGmUrAmUrAmGrUmCrGC-3′; antisense5′-/5Phos/rGrCrGrArCrUrArUrArCrGrCrGrCrArArUrArUmGmG rU-3′; IDT) were reverse transfected at 2 nM using Lipofectamine RNAiMax (Life Technologies) according to manufacturer guidelines.

### 2.8. Chromatin Immunoprecipitation (ChIP)

ChIP assay was conducted using MAGnify™ Chromatin Immunoprecipitation System (Thermo Fisher Scientific, #49-2024, Grand Island, NY, USA) according to manufacturer directions. Untreated OVCAR8, OVSAHO, MCF-7 cells with or without 10 ng/mL of TGFB1 were crosslinked with 1% formaldehyde. 1.25 M glycine in cold PBS were then added to stop the crosslinking reaction. Cell lysates were prepared with lysis buffer with protease inhibitors (50 μL per 1 million cells). Chromatin was then sheared into 200–500-bp fragments using Sonic Dismembrator Model F60 With Probe (Thermo Fisher Scientific, Grand Island, NY, USA). Each immunoprecipitation (IP) reaction contains 100,000 cells. Dynabeads^®^ (Thermo Fisher Scientific, Grand Island, NY, USA) were coupled with anti-Snail (L70G2; Cell Signaling Technology, Danvers, MA, USA) or Mouse IgG (supplied in MAGnify kit) as negative controls (1 μg per CHIP). After 1 h on a rotor, these antibody-Dynabeads^®^ complexes were incubated with chromatin and put on rotor for 2 h at 4 °C. As input control, 10 μL of diluted chromatin were put aside without binding to the antibody-Dynabeads^®^ complexes. After chromatin-antibody-Dynabeads^®^ complexes were washed with IP buffer to remove unbound chromatin. Reverse Crosslinking buffer was added to reverse the formaldehyde crosslinking. Real-time RT-qPCR for DNA was performed using PowerUP SYBR Green master mix (Thermo Fisher Scientific, Grand Island, NY, USA) and specific primers on a Stratagene Mx3005P instrument (Agilent Technology, Santa Clara, CA, USA). Primer sequences are listed in Table S3. The results were analyzed using the ΔΔ cycles to threshold (ΔΔCt) method; ACTB was used for normalization.

### 2.9. Luciferase Assays

HEK293T cells were plated at 50,000 cells per well. Twenty-four hours later PEI reagent was used to transfect cells with 200 ng full length *let-7*, truncated *let-7i* (*lucB*), or mutated *let-7i* (*mlucB*) promoter luciferase vector in combination with 5 ng Renilla luciferase, and 200 ng *SNAI1*-expressing or empty vector (Addgene 16218). Forty-eight hours post transfection (or twenty-four hours for promoter truncation/mutation) dual-luciferase reporter assay kit (Promega, San Luis Obispo, CA, USA) was used to analyze bioluminescence on SpectraMax i3x microplate reader (Molecular Devices, Sunnyvale, CA, USA). *Let-7a1df1* promoter luciferase was a kind gift from Dr. Zifeng Wang [50], *let-7a3* from Dr. Hillary Coller [51], *Let-7c* from Dr. Maria Rizzo [52,53], full length *let-7i* from Dr. Steve O’Hara [54], and truncated (lucB)/mutated (mlucB) *let-7i* from Dr. Muh-Hwa Yang [33].

### 2.10. Spheroid Formation Assay

Cells were plated at a density of 10,000 cells/mL (12,000 cells/mL for PDX6 cells) in non-tissue culture coated plates, 10 technical replicates per condition, and maintained in serum-free medium (DMEM/F12 50/50) supplemented with 0.4% bovine serum albumin, 10 ng/mL FGF, 20 ng/mL EGF, 6.7 ng/mL selenium, 5.5 µg/mL transferrin, 10 µg/mL insulin, and 1% knock out serum replacement (Gibco/Thermo Fisher Scientific) for 7 days. Secondary spheroid assays were done by harvesting after seven days, trypsinization, and re-seeding at 10,000 cells/mL, followed by seven additional days of growth. To determine the number and size of spheroids, phase contrast images of spheroids taken on a Nikon Eclipse Ti microscope were analyzed using ImageJ software (National Institutes of Health, Bethesda, MD, USA).

### 2.11. Mice

All animal procedures were conducted according to animal care guidelines approved by the Institutional Animal Care and Use Committee at Loma Linda University (IACUC #8170044, 9 November 2017). Orthotopic PDX experiments were carried out in nude mice (nu/nu), obtained from Jackson Laboratory (Sacramento, CA, USA), which were housed in specific pathogen-free conditions, and were used for xenografts at 6–10 weeks of age.

### 2.12. Orthotopic Xenograft Model and Live Animal Imaging

To allow in vivo visualization, PDX6 cells were transduced with a CMV-p:EGFP-ffluc pHIV7 lentiviral vector (eGFP-ffluc, kind gift of Christine Brown, City of Hope, Duarte, CA USA) [55], which encodes a fusion protein of GFP and firefly luciferase. The eGFP-ffluc-transduced PDX6 cells were selectively isolated based on GFP expression via FACSAria cell sorter (BD Biosciences, San Jose, CA, USA). PDX6 cells were injected into the right ovarian bursa of nude mice with Matrigel (354248; Corning, Corning, NY, USA) at 2.5 × 10^5^ cells per mouse, eight mice per condition. For in vivo experiments, DsiRNA with 2′-O-methyl modifications were used [56] (modified oligonucleotide sequence listed Table S4). Starting 1 week after initial injection and continuing twice weekly, HA-MSN-siRNA were injected intravenously. After intraperitoneal injection of luciferin, the mice were imaged with an IVIS Lumina Series III in vivo imaging system (PerkinElmer, Waltham, MA, USA). Live imaging was performed twice weekly and the bioluminescent images were analyzed using Living Image in vivo Imaging Software (PerkinElmer, Waltham, MA, USA) to assess tumor burden at primary and metastatic sites. At day 1, 14 mice were randomized and assigned into two groups (siControl and siSnail, 7 mice each). The bioluminescence of animals from each group was measured at each time point. Based on tumor development, some mice were censored from analyses. Each animal’s measurement was normalized to its own bioluminescence from day one and then the means for each time point were analyzed using a two-way ANOVA. To determine endpoints, mouse abdominal girth was measured prior to surgery and monitored once a week. When the first mouse reached the endpoint of an increase of 25% in girth, all mice were euthanized, and necropsy was carried out. Primary and metastatic tumor weight and tumor locations were recorded, and samples were harvested for gene and protein expression analysis.

### 2.13. Statistical Analyses

For all in vitro experiments, cell samples in the same treatment group were harvested from at least 3 biological replicates and processed individually. For in vivo experiments, data are from one representative experiment of three. All values in the figures and text are the means ± SD. Statistical analyses were performed using the Prism 7.0a for Mac OS X (GraphPad Software, Inc., San Diego, CA, USA). Statistical significance among mean values was determined by Student’s t-test with two-tailed alpha level of 0.05 considered significant, with the exception of tumor growth in the in vivo study, which is determined by two-way ANOVA with Tukey’s multiple comparison test. * *p* < 0.05; ** *p* < 0.01; *** *p* < 0.001; **** *p* < 0.0001.

## 3. Results

### 3.1. SNAI1 Leads to Increased Stemness

To test the relationship between *SNAI1* expression and changes in stemness, we induced *SNAI1* expression with growth factors including TGFB1 and EGF [57,58]. We tested several cancer cell lines of epithelial origin including pancreatic (PANC-1), breast (MCF-7), and ovarian (OVCAR8 and OVSAHO).

**Figure 1:**
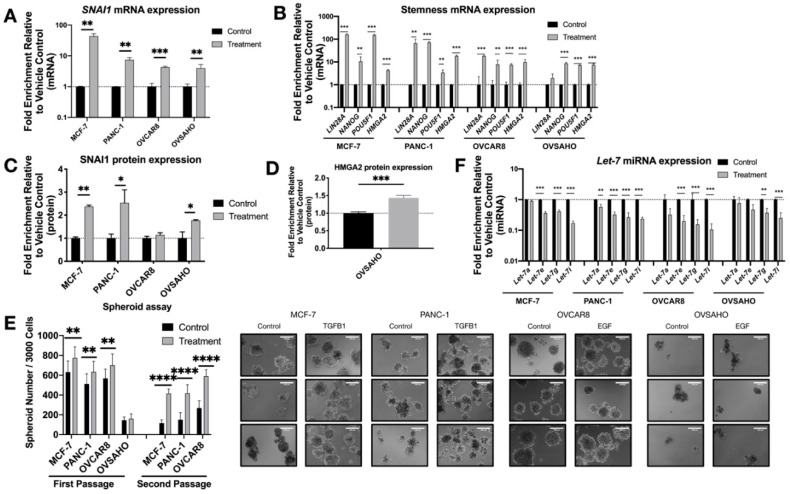
Growth factor treatment results in increased SNAI1, increased stemness and decreased *let-7* expression. MCF-7, PANC-1 were treated with TGFB1; OVCAR8, OVSAHO were treated with EGF. Levels of control group (cells treated with vehicle control) were normalized to 1. Values for RT-qPCR are shown on a log scale. (**A**,**B**) RT-qPCR analysis for mRNA expression level of *SNAI1* (**A**) and of stemness markers ((**B**), *LIN28A, NANOG, POU5F1* and *HMGA2*). (**C**,**D**) The quantification of Western blot analysis for protein expression of SNAI1 (**C**) and HMGA2 (**D**). (**E**) Left panel: The quantification of number of spheroids per 3000 cells (both first passage and second passage) is shown. Right panel: Phase contrast images of spheroids formed from cells (first passage) as indicated are presented. In each panel, the spheroids formed from control group are presented on the left, those from the treatment group are on the right. Scale bar = 100 μm (**F**) RT-qPCR analysis for *let-7* miRNA (*let-7a*, *let-7e*, *let-7g* and *let-7i)* expression. * *p* < 0.05; ** *p* < 0.01; *** *p* < 0.001; **** *p* < 0.0001.

After two days of TGFB1 (MCF-7 or PANC-1) or EGF (OVSAHO or OVCAR8) treatment, as expected, RNA and protein expression levels of *SNAI1* increased modestly, confirmed by RT-qPCR (Figure 1A) and Western blot (Figure 1C and Figure S2A). TGFB1 does not induce *SNAI1* expression in OVSAHO or OVCAR8 (Figure S1B); for this reason, ovarian cancer cell lines were treated with EGF. The smaller change in SNAI1 protein observed in OVCAR8 could be explained by its high endogenous *SNAI1* level as previously described [59]. Endogenous levels of all cell lines are shown in Figure S1A. mRNA expression of stemness markers *LIN28A*, *NANOG, POU5F1* and *HMGA2* increased after treatment (Figure 1B). Western blot analysis showed an increase of HMGA2 protein in OVSAHO (43%) (Figure 1D and Figure S2B). However, this was not detectable in other lines. We used spheroid assays as a measure of self-renewal and growth in non-adherent conditions, which are increased with higher stemness [59,60]. In agreement with the phenotypic measurements above, cells in which *SNAI1* expression was induced via TGFB1 or EGF formed more spheroids, to a greater extent in secondary spheroids (Figure 1E and Figure S5B). Along with the increased *SNAI1* expression, consistent with a change to a more stem cell-like gene expression pattern, we observed a decrease in expression levels of *let-7* family members (Figure 1F). We chose to follow one *let-7* member from each of four clusters on chromosomes 3, 9, 12, and 19 [23].

Because growth factor-induced EMT resulted in changes consistent with increased stemness, we wished to pinpoint mechanisms of stemness downstream of EMT. Our previous studies indicated a role for *SNAI1* in the induction of the stem cell fate [17]. Besides inducing EMT, the TGFB1 signaling pathway is important in mediating cellular proliferation, preventing progression through the cell cycle, and multiple other actions [57]. EGF also plays an important role in the development of tumors by regulating cell proliferation, differentiation, migration and angiogenesis [58]. Thus, treatment with these growth factors changes the expression of numerous genes besides *SNAI1*. To specify the effect of a single factor, *SNAI1*, we overexpressed *SNAI1* to determine whether it alone could induce the stem cell state. Cell lines were virally transduced with constitutively expressed *SNAI1* or control vector.

**Figure 2:**
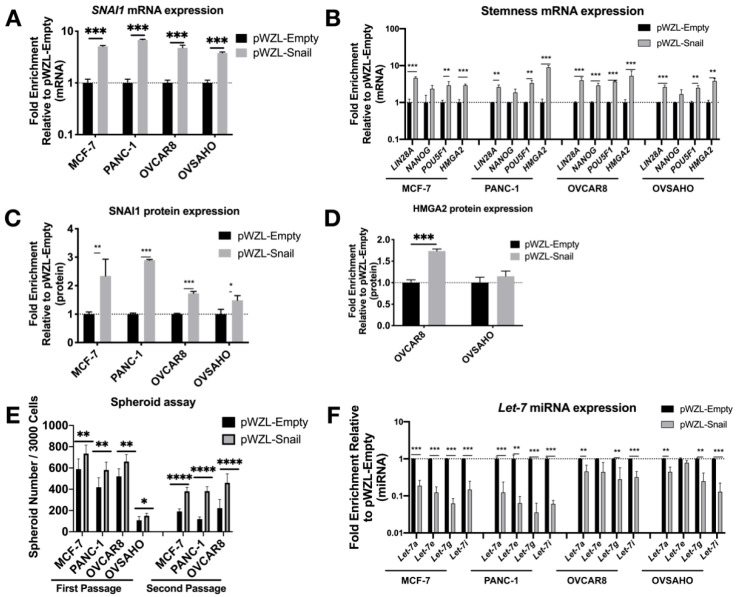
*SNAI1* overexpression results in increased stemness and decreased *let-7* expression. Cell lines were transduced with the retroviral expression vector pWZL-Snail or empty vector, pWZL-Empty, in cell lines MCF-7, PANC-1, OVCAR8 and OVSAHO. Levels of control group (cells transduced with pWZL-Empty) were normalized to 1. Values for RT-qPCR are shown on a log scale. (**A**,**B**) RT-qPCR analysis for mRNA expression of *SNAI1* (**A**) and of stemness markers *LIN28A*, *NANOG*, *POU5F1* and *HMGA2* (**B**). (**C**,**D**) The quantification of Western blot analysis for protein expression of SNAI1 (**C**) and HMGA2 (**D**). (**E**) The quantification of number of spheroids formed per 3000 cells (both first passage and second passage) as indicated. (**F**) RT-qPCR analysis for *let-7* miRNA (*let-7a*, *let-7e*, *let-7g* and *let-7i)* expression. * *p* < 0.05; ** *p* < 0.01; *** *p* < 0.001; **** *p* < 0.0001

After transduction, the increase in *SNAI1* mRNA and protein expression (Figure 2A,C and Figure S4A) was accompanied by a significant increase in stemness markers *LIN28A*, *POU5F1*, and *HMGA2* (Figure 2B). Western blot data confirmed this change, showing an increase in HMGA2 (Figure 2D and Figure S4B). With the increase in expression of *SNAI1* and stemness genes, we observed a decrease in *let-7* family members (Figure 2F). Consistent with the phenotypic changes, *SNAI1* overexpression led to an increased number of spheroids formed, to a greater extent in secondary spheroids (Figure 2E, Figures S4C and S5B) (the size of spheroids for OVCAR8 is quantified and presented in Figure S5A). These results suggest increased stemness associated with *SNAI1*. In order to investigate whether the regulation of stemness is directly through SNAI1’s action on *let-7*, we overexpressed *let-7i* in *SNAI1* overexpressing cells (Figure S6A). *Let-7i* overexpression resulted in abrogation of *SNAI1*-induced stemness as measured by RT-qPCR (Figure S6B) and spheroid formation (Figure S6C,D). These results are consistent with the observation that *SNAI1* overexpression is sufficient to shift the phenotype toward stemness via its effect on *let-7*.

### 3.2. SNAI1 Knockdown Reverses Stemness

**Figure 3:**
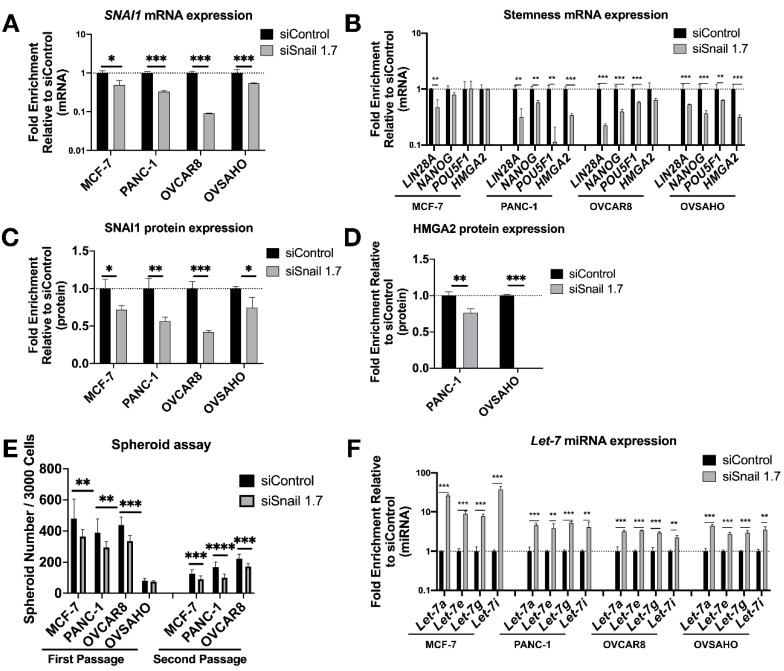
*SNAI1* knockdown reverses stemness and restores *let-7* expression. Mesoporous silica nanoparticles coated with hyaluronic acid (HA-MSN) were used to deliver siRNA (siSnail and siControl) in MCF-7, PANC-1, OVCAR8, and OVSAHO. Levels of control group (cells treated with siControl) were normalized to 1. Values for RT-qPCR are shown on a log scale. Samples were harvested after 24 h (MCF-7, OVCAR8 and OVSAHO) or 72 h (PANC-1). (**A**,**B**) RT-qPCR analysis for mRNA expression of SNAI1 (**A**) and of stemness markers *LIN28A*, *NANOG*, *POU5F1* and *HMGA2* (**B**). (**C**,**D**) The quantification of Western blot analysis for protein expression of SNAI1 (**C**) and HMGA2 (**D**). (**E**) The quantification of number of spheroids formed per 3000 cells (both first passage and second passage) as indicated. (**F**) RT-qPCR analysis for *let-7* miRNA (*let-7a*, *let-7e*, *let-7g*, and *let-7i*) expression. * *p* < 0.05; ** *p* < 0.01; *** *p* < 0.001; **** *p* < 0.0001

Having established the impact of *SNAI1*’s gain-of-function on cells’ stemness and *let-7* levels, we proceeded to knock down *SNAI1* to test if the opposite effects could be observed. We used HA-conjugated MSN [45] (HA-MSN) to deliver siRNA in MCF-7, PANC-1, OVSAHO and OVCAR8. We observed a decrease in the mRNA expression level of *SNAI1* after HA-MSN-siSnail treatment in most cases (Figure 3A). The knockdown of *SNAI1* was confirmed on the protein level with Western blot data (Figure 3C and Figure S7A). Together with the decrease of *SNAI1*, the expression of stemness markers also decreased on the mRNA level (Figure 3B). HMGA2 protein also decreased in PANC-1 and OVSAHO after siSnail treatment (Figure 3D and Figure S7B). *SNAI1* knockdown resulted in reduced frequency of stem cells, as measured by number of spheroids formed (Figure 3E and Figure S7C), and secondary spheroids showed a greater difference between siSnail and siControl (Figure 3E and Figure S5B). An increase in spheroid size in OVCAR8 was also observed (Figure S5A). Consistent with the *SNAI1* time course, *let-7* expression increased after *SNAI1* knockdown (Figure 3F). Similar effects can be observed with a different siRNA (Figure S8). These results indicate that reducing *SNAI1* expression leads to decreased stemness and the restoration of *let-7* expression in cancer cells.

### 3.3. SNAI1 Knockdown Reverses Stemness in Patient Derived HGSOC Samples In Vitro and Decreases Tumor Burden In Vivo

**Figure 4:**
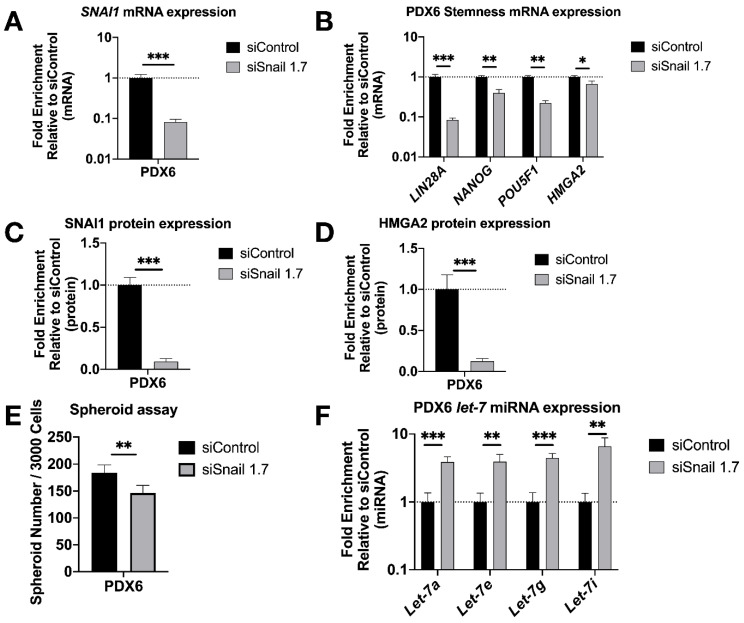
*SNAI1* knockdown reduces stemness in patient-derived cells in vitro. HA-MSN were used to deliver siRNA (siSnail and siControl) in PDX cells in vitro. Levels of control group (cells treated with siControl) were normalized to 1. Values for RT-qPCR are shown on a log scale. (**A**,**B**) RT-qPCR analysis for mRNA expression of SNAI1 (**A**) and of stemness markers *LIN28A*, *NANOG*, *POU5F1* and *HMGA2* (**B**). (**C**,**D**) The quantification of Western blot analysis for protein expression of SNAI1 (**C**) and HMGA2 (**D**). (**E**) The quantification of number of spheroids per 3000 cells formed from PDX6 in vitro. (**F**) RT-qPCR analysis for *let-7* miRNA (*let-7a*, *let-7e*, *let-7g,* and *let-7i*) expression. * *p* < 0.05; ** *p* < 0.01; *** *p* < 0.001.

To test our findings in a more clinically relevant setting, we knocked down *SNAI1* in patient-derived HGSOC cells in vitro using HA-MSN-siSnail (Figure 4A,C and Figure S9A). In agreement with our observations in cell lines, PDX cells treated with HA-MSN-siSnail showed decreased levels of stemness markers (Figure 4B,D and Figure S9A), decreased size (Figure S5A) and number of spheroids formed (Figure 4E and Figure S9B), and increased levels of *let-7* (Figure 4F).

**Figure 5:**
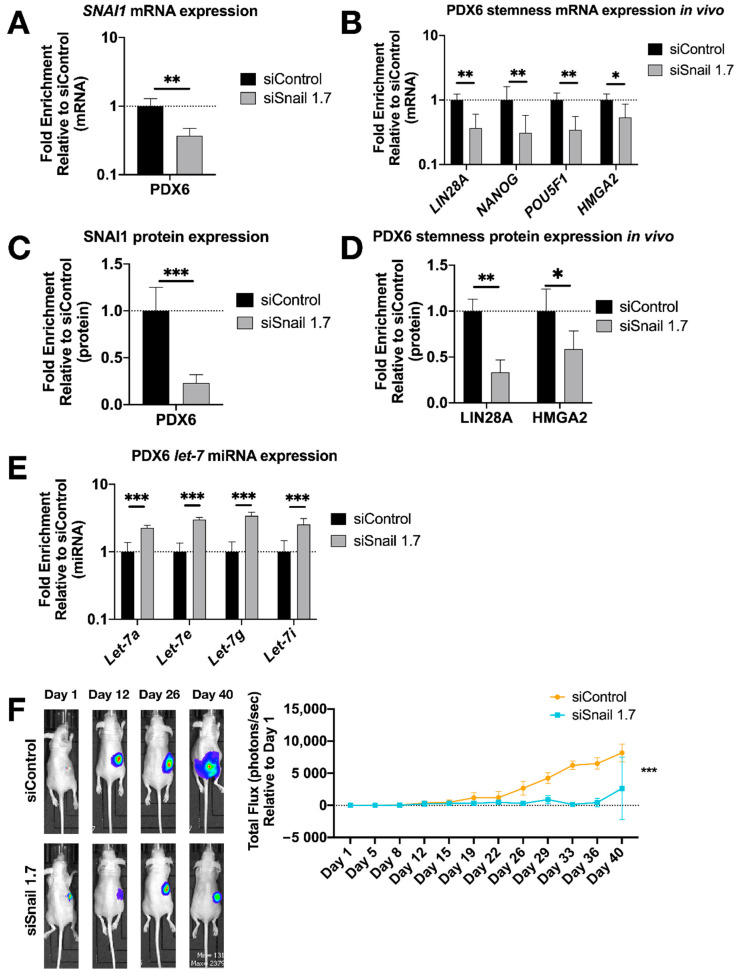
*SNAI1* knockdown in vivo reduces stemness gene expression and tumor burden. HA-MSN were used to deliver siRNA (siSnail and siControl) via IV injection to orthotopic PDX in vivo. Tumor samples were harvested and analyzed at necropsy. Levels of control group (cells treated with siControl) were normalized to 1. Values for RT-qPCR are shown on a log scale. (**A**,**B**) RT-qPCR analysis for mRNA expression of *SNAI1* (**A**) and of stemness markers *LIN28A*, *NANOG*, *POU5F1* and *HMGA2* (**B**), in tumors. (**C**,**D**) The quantification of Western blot analysis for protein expression of SNAI1 (**C**) and stemness markers LIN28A and HMGA2 (**D**), in tumors. (**E**) RT-qPCR analysis for *let-7* miRNA (*let-7a*, *let-7e*, *let-7g* and *let-7i*) expression in tumors. (**F**) Left panel: Representative images of xenograft mice. siControl (upper) and siSnail knockdown (lower). Right panel: Quantitation of bioluminescence at primary sites over six weeks. X axis, days; Y axis, total flux in photons/second relative to day 1. * *p* < 0.05; ** *p* < 0.01; *** *p* < 0.001.

To extend these results to an in vivo setting, luciferized PDX6 cells were injected into the ovarian bursa of nude mice in our orthotopic xenograft model [59]. Mice were imaged twice weekly for bioluminescence, and total flux was quantified over seven weeks. One week after bursa injection, treatment with HA-MSN-siSnail (or HA-MSN-siControl) began and continued twice weekly for the duration of the experiment. Upon necropsy, RT-qPCR results showed a decrease of *SNAI1* along with reduced *LIN28A, NANOG and POU5F1* in tumors from mice treated with HA-MSN-siSnail (Figure 5A,B). In agreement with mRNA results, the protein levels of SNAI1, LIN28A and HMGA2 were significantly decreased in mice treated with HA-MSN-siSnail (Figure 5C,D and Figure S10A). Consistent with the in vitro results, *let-7* levels were also increased in mice treated with HA-MSN-siSnail (Figure 5E). In addition, primary tumor weights demonstrated smaller tumors in siSnail mice (Figure S10B). Visualization of tumors in live animals revealed that primary tumors were significantly smaller in mice receiving HA-MSN-siSnail injections (Figure 5F). These results demonstrate that *SNAI1* was successfully knocked down in vivo using targeted nanoparticle-delivered RNAi. Taken together, our results demonstrate that knockdown of *SNAI1* in patient-derived HGSOC samples in vitro and in vivo results in restoration of *let-7*, decreased stemness, and reduced tumor burden.

### 3.4. SNAI1 Binds let-7 Promoters Resulting in let-7 Repression

**Figure 6:**
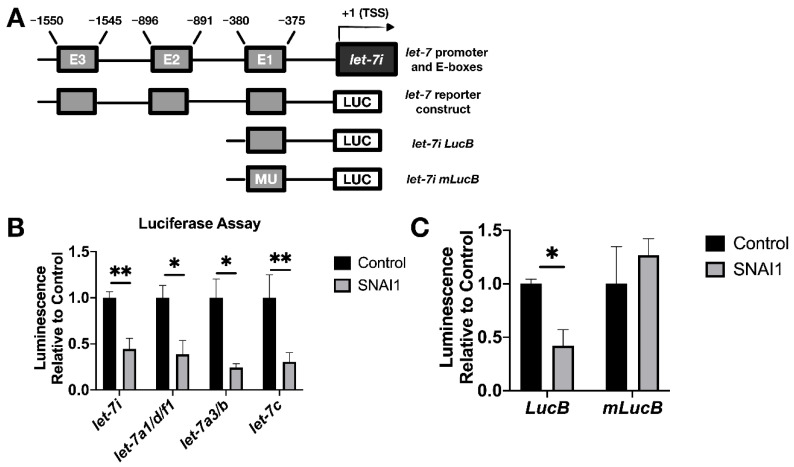
*SNAI1* represses *let-7* promoters. (**A**) Schematic representation of the promoter region of *let-*7*i* (upper) and reporter constructs used in luciferase assays (lower diagrams). E1, E2, E3: E-boxes (sequence: CANNTG); MU: Mutated E-boxes; TSS: Transcription start site (**B**) For luciferase assays, HEK293T cells were co-transfected with two plasmids: (1) *let-7* promoter luciferase *(let-7i, let-7a1/d/f1, let-7a3/b, let-7c)*, and (2) either *SNAI1* (constitutively expressed, gray bars) or empty vector (black bars). Luminescence activity was measured 48 h thereafter. (**C**) HEK293T cells were co-transfected with either *let-7i* lucB or *let-7i* mlucB with or without *SNAI1.* Luminescence was measured 24 h later. * *p* < 0.05; ** *p* < 0.01.

We sought to establish whether *SNAI1* acts to repress *let-7* transcription directly. *SNAI1* binds promoters of *let-7* in fibroblasts, and binding increases upon *SNAI1* overexpression [17]. To examine whether this same association can be observed in cancer cells, we carried out ChIP assays to determine the binding of *SNAI1* to the promoter region of various *let-7* family members, as defined by previous studies [33,50,51,52,53,54]. The *let-7i* promoter is diagrammed in Figure 6A; [33,54] the promoter region locations and the E-box (CANNTG) locations studied are listed in Table S1. At baseline, we observed that *SNAI1* bound *CDH1* (used as a positive control) and *let-7* promoters to a greater extent in OVCAR8, the cell line with higher *SNAI1* expression, than in OVSAHO [59] (Figure S11A). We also assessed binding upon EMT induction by TGFB1 in MCF-7 cells and detected an increased level of *let-7i* and *miR-98* promoter binding compared to the control group (Figure S11B). These data demonstrate *SNAI1* binding to *let-7* promoter regions in cancer cells tested.

To test the functional result of *SNAI1* binding to *let-7* promoters, luciferase assays were used as a reporter for *let-7* promoter activity via bioluminescence. We used let-7 promoter luciferase constructs as shown in Figure 6A (bottom diagram; see Table S1). This enabled us to detect the effect of *SNAI1* on *let-7i, let7a1/d/f1, let-7a-3,* and *let-7c* promoter activity. Co-transfection with *let-7* promoter luciferase and *SNAI1* (constitutively expressed), compared with empty vector, resulted in a reduction in bioluminescence (Figure 6B), confirming the repression of *let-7* promoter activity. Expression from a truncated promoter containing only E-box one was also reduced by overexpressed *SNAI1* (E-boxes are the binding site for *SNAI1*). However, when the same E-box was mutated, the inhibition by *SNAI1* was abrogated (Figure 6C). These results demonstrate that *SNAI1* binding to *let-7* promoters directly represses *let-7* transcription.

## 4. Discussion

*Let-7*’s major roles in maintenance of differentiation make it a key player in both development and cancer [13,14]. Loss of *let-7* is a major component of the loss of differentiation seen in many cancers, and significantly correlates with poor prognosis [13,16,18,19]. Studies of stem cell reprogramming linked *let-7* repression with a transcription factor that induces EMT, *SNAI1* [17]. In the present study, we examined the role of *let-7* in cancer cells and its connection to *SNAI1*. When cells from breast (MCF-7), pancreatic (PANC-1), and ovarian (OVCAR8, OVSAHO) cancer were treated with EMT-inducing agents (TGFB1 or EGF), increases in EMT factors including *SNAI1*, increases in stemness markers, and decreases in *let-7* could be detected. This positive association between *SNAI1* and stemness, and the negative association between *SNAI1* and *let-7*, were confirmed when *SNAI1* itself was overexpressed through viral transduction or knocked down by siRNA.

One of the goals of this investigation was to understand the molecular mechanisms by which *SNAI1* exerts its pro-stemness effects. The effect of *SNAI1* on *let-7* levels, and its direct binding to several *let-7* family member promoter regions, were detected using ChIP and luciferase assays, providing evidence that *SNAI1* binds *let-7* promoters and directly represses its expression, leading to an increase in stemness in cancer cells. Although EMT has been linked to stemness, few insights into downstream mechanisms have been generated. One downstream effector of *SNAI1* and other EMT programs is the transcription factor FOXC2 via the serine/threonine kinase p38, thus linking EMT and stem cell traits [39]. Another avenue by which *SNAI1* exerts stemness is via repression of miR-34 via effects on WNT signaling, NOTCH, and CD44 [61]. Presented results provide evidence for the *SNAI1*/*let-7* axis as a crucial mechanism by which EMT exerts pro-stemness roles. These results point to *SNAI1* as a stem cell-directed target for therapy.

*SNAI1* may be a particularly apt target in the goal of eliminating CSC because of its role in the stabilization of the hybrid epithelial–mesenchymal state [37,62]. OVCAR8 parental cells showed the highest level of stemness markers (*LIN28A*, *NANOG*, *POU5F1* and *HMGA2)*, along with a high level of epithelial marker *CDH1* and mesenchymal markers *SNAI1* and *VIM* (Figure S1A), consistent with a hybrid EMT status. LIN28/*let-7* circuits regulate stemness as shown in both modeling and in vitro experiments [32,63]. Cells have been categorized on this basis into differentiated (d), d/u hybrid or undifferentiated (u) states, based on LIN28 level. Significant correspondence between E/M state to D/U hybrid state has been theoretically demonstrated [32]. Therefore, the hybrid EMT state has been proposed to be most likely to gain stemness via *let-7* regulation. *SNAI1* is highly expressed in all of the cell types examined here (Figure S1A), and further studies will determine whether *SNAI1*-dependent *let-7* repression plays a role in the hybrid state.

*SNAI1* inhibition via transfection, viral delivery, or genetic deletion has been shown to reduce invasion, proliferation, chemoresistance, and other aspects of the stemness phenotype [59,64,65]. However, because these approaches cannot be considered for use in patients, novel approaches like the nanoparticle-mediated delivery are needed. Small RNAs can be efficiently loaded onto MSNs, which protect the oligonucleotides from degradation, are enriched in tumors due to leaky vasculature, and are taken up into cells by pinocytosis, in effect functioning as a transfection reagent [46]. Their large surface area and pore structure make them ideal for drug delivery [44]. MSNs are a promising delivery agent for RNAi in vivo [45,49,66]. Considering this potential, and with the goal of clinical relevance, we used MSN to knock down *SNAI1*. *SNAI1* downregulation could be detected on both RNA and protein levels, emphasizing the utility of MSN for siRNA delivery. We extended these results to in vivo experiments where we knocked down *SNAI1* in our orthotopic PDX model [59]. We achieved >75% knockdown of SNAI1 protein in tumors in vivo. Furthermore, tumor *let-7* levels increased 2–3 fold, consistent with *SNAI1*-mediated repression of *let-7* in vivo. In parallel, expression of stem cell markers *LIN28A*, *NANOG*, *POU5F1*, and *HMGA2* decreased, consistent with a shift away from the stem cell phenotype. This demonstrates that targeting *SNAI1* is sufficient to reduce stemness. Further studies will determine if these changes lead to reduced metastasis or delayed recurrence.

Although these studies provide important insights into the mechanism for loss of *let-7* and thus the destabilization of the differentiated state, they do not address the question of the origin of CSC. Rather, we suggest that any cell, regardless of origin, will lose *let-7* while taking on the characteristics of cancer stem cells. Like differentiated cells, adult stem cells express high levels of *let-7* [67,68], therefore *let-7* loss via transcriptional, post-transcriptional, or epigenetic regulation is required even if adult stem cells are the cell of origin. In the absence of *LIN28A*, transcriptional repression of *let-7* could tip the balance in favor of stemness. The mechanism by which *let-7* is lost is thus germane to cancer stem cell biology regardless of whether normal stem cells or differentiated cells are the cells of origin. Our finding that *SNAI1* transcriptionally represses *let-7* adds even more weight to *SNAI1* as a therapeutic target. Blocking *SNAI1*, in addition to inhibiting invasion and migratory ability, is expected to restore *let-7* by increasing its transcription. We predict that *SNAI1*-mediated *let-7* repression could be an important mechanism of cancer stemness in a wide variety of carcinoma cells.

## 5. Conclusions

Our studies reveal one mechanism for EMT-induced stemness. *SNAI1* overexpression results in reduction of miRNA *let-7* levels, and is sufficient to shift the phenotype of cancer cells tested toward stemness. *SNAI1* knockdown leads to decreased stemness and the restoration of *let-7* expression in cancer cells, including patient-derived cells. ChIP and luciferase assays led to the conclusion that *SNAI1* binding to *let-7* promoters directly represses *let-7* transcription. Mesoporous silica nanoparticle-delivered RNAi effectively knocks down *SNAI1* in vivo, resulting in reduced tumor burden, in support of its clinical use. These results provide evidence for the *SNAI1*/*let-7* axis as a key mechanism by which EMT exerts pro-stemness roles.

## Data Availability

Data will be made available from the corresponding author upon reasonable request.

## Supplementary Materials

The following are available online at https://www.mdpi.com/2072-6694/13/6/1469/s1, Figure S1: Parental cell line gene expression comparison; EMT is not induced by TGFB1 treatment in ovarian cancer cell lines, Figure S2: Western blot data for growth factor treatment, Figure S3: The effectiveness of different DsiRNAs, Figure S4: Western blot data and pictures of spheroids formed with SNAI1 overexpression, Figure S5: Higher level of *SNAI1* is associated with larger size of spheroids and higher number of secondary spheroids formed, Figure S6: *Let-7i* overexpression rescues the increase in stemness resulting from *SNAI1* overexpression, Figure S7: Western blot data and pictures of spheroids formed in SNAI1 knockdown in cancer cell lines, Figure S8: Knockdown of *SNAI1* with a second DsiRNA, Figure S9: Western blot data and pictures of spheroids formed in SNAI1 knockdown in PDX cells in vitro, Figure S10: Western blot data and tumor weight comparison for *SNAI1* knockdown in PDX cells in vivo, Figure S11: SNAI1 binds to *let-7* promoters demonstrated by ChIP, Figure S12: Uncropped Western blot figures, Table S1: *Let-7* promoter region length, locations and E-box (CANNTG) locations, Table S2: Sequences of primers used in RT-qPCR, Table S3: Sequences of primers used in chromatin immunoprecipitation, Table S4: Sequences of siRNA oligonucleotides.

## References

[1] Kreso A., Dick J.E. (2014). Evolution of the Cancer Stem Cell Model. Cell Stem Cell.

[2] Al-Hajj M., Wicha M.S., Benito-Hernandez A., Morrison S.J., Clarke M.F. (2003). Prospective Identification of Tumorigenic Breast Cancer Cells. Proc. Natl. Acad. Sci. USA.

[3] Reya T., Morrison S.J., Clarke M.F., Weissman I.L. (2001). Stem Cells, Cancer, and Cancer Stem Cells. Nature.

[4] Yamada Y., Haga H., Yamada Y. (2014). Concise Review: Dedifferentiation Meets Cancer Development: Proof of Concept for Epigenetic Cancer. Stem Cells Transl. Med..

[5] Schwitalla S., Fingerle A.A., Cammareri P., Nebelsiek T., Göktuna S.I., Ziegler P.K., Canli O., Heijmans J., Huels D.J., Moreaux G. (2013). Intestinal Tumorigenesis Initiated by Dedifferentiation and Acquisition of Stem-Cell-like Properties. Cell.

[6] Vermeulen L., De Sousa E., Melo F., van der Heijden M., Cameron K., de Jong J.H., Borovski T., Tuynman J.B., Todaro M., Merz C. (2010). Wnt Activity Defines Colon Cancer Stem Cells and Is Regulated by the Microenvironment. Nat. Cell Biol..

[7] Friedmann-Morvinski D., Verma I.M. (2014). Dedifferentiation and Reprogramming: Origins of Cancer Stem Cells. EMBO Rep..

[8] Daley G.Q. (2008). Common Themes of Dedifferentiation in Somatic Cell Reprogramming and Cancer. Cold Spring Harb. Symp. Quant. Biol..

[9] Riggs J.W., Barrilleaux B.L., Varlakhanova N., Bush K.M., Chan V., Knoepfler P.S. (2013). Induced Pluripotency and Oncogenic Transformation Are Related Processes. Stem Cells Dev..

[10] Kim J., Orkin S.H. (2011). Embryonic Stem Cell-Specific Signatures in Cancer: Insights into Genomic Regulatory Networks and Implications for Medicine. Genome Med..

[11] Gupta P.B., Chaffer C.L., Weinberg R.A. (2009). Cancer Stem Cells: Mirage or Reality?. Nat. Med..

[12] Ben-Porath I., Thomson M.W., Carey V.J., Ge R., Bell G.W., Regev A., Weinberg R.A. (2008). An Embryonic Stem Cell-like Gene Expression Signature in Poorly Differentiated Aggressive Human Tumors. Nat. Genet..

[13] Boyerinas B., Park S.-M., Hau A., Murmann A.E., Peter M.E. (2010). The Role of Let-7 in Cell Differentiation and Cancer. Endocr. Relat. Cancer.

[14] Büssing I., Slack F.J., Grosshans H. (2008). Let-7 MicroRNAs in Development, Stem Cells and Cancer. Trends Mol. Med..

[15] Boyerinas B., Park S.-M., Shomron N., Hedegaard M.M., Vinther J., Andersen J.S., Feig C., Xu J., Burge C.B., Peter M.E. (2008). Identification of Let-7-Regulated Oncofetal Genes. Cancer Res..

[16] Park S.-M., Shell S., Radjabi A.R., Schickel R., Feig C., Boyerinas B., Dinulescu D.M., Lengyel E., Peter M.E. (2007). Let-7 Prevents Early Cancer Progression by Suppressing Expression of the Embryonic Gene HMGA2. Cell Cycle Georget. Tex.

[17] Unternaehrer J.J., Zhao R., Kim K., Cesana M., Powers J.T., Ratanasirintrawoot S., Onder T., Shibue T., Weinberg R.A., Daley G.Q. (2014). The Epithelial-Mesenchymal Transition Factor SNAIL Paradoxically Enhances Reprogramming. Stem Cell Rep..

[18] Chirshev E., Oberg K.C., Ioffe Y.J., Unternaehrer J.J. (2019). Let-7 as Biomarker, Prognostic Indicator, and Therapy for Precision Medicine in Cancer. Clin. Transl. Med..

[19] Shell S., Park S.-M., Radjabi A.R., Schickel R., Kistner E.O., Jewell D.A., Feig C., Lengyel E., Peter M.E. (2007). Let-7 Expression Defines Two Differentiation Stages of Cancer. Proc. Natl. Acad. Sci. USA.

[20] Viswanathan S.R., Daley G.Q., Gregory R.I. (2008). Selective Blockade of MicroRNA Processing by Lin28. Science.

[21] Baum B., Settleman J., Quinlan M.P. (2008). Transitions between Epithelial and Mesenchymal States in Development and Disease. Semin. Cell Dev. Biol..

[22] Chirshev E., Hojo N., Bertucci A., Sanderman L., Nguyen A., Wang H., Suzuki T., Brito E., Martinez S.R., Castañón C. (2020). Epithelial/Mesenchymal Heterogeneity of High-Grade Serous Ovarian Carcinoma Samples Correlates with MiRNA Let-7 Levels and Predicts Tumor Growth and Metastasis. Mol. Oncol..

[23] Lee H., Han S., Kwon C.S., Lee D. (2016). Biogenesis and Regulation of the Let-7 MiRNAs and Their Functional Implications. Protein Cell.

[24] Micalizzi D.S., Farabaugh S.M., Ford H.L. (2010). Epithelial-Mesenchymal Transition in Cancer: Parallels between Normal Development and Tumor Progression. J. Mammary Gland Biol. Neoplasia.

[25] Nieto M.A. (2013). Epithelial Plasticity: A Common Theme in Embryonic and Cancer Cells. Science.

[26] Pastushenko I., Blanpain C. (2019). EMT Transition States during Tumor Progression and Metastasis. Trends Cell Biol..

[27] Bocci F., Jolly M.K., Tripathi S.C., Aguilar M., Hanash S.M., Levine H., Onuchic J.N. (2017). Numb Prevents a Complete Epithelial-Mesenchymal Transition by Modulating Notch Signalling. J. R. Soc. Interface.

[28] Battula V.L., Evans K.W., Hollier B.G., Shi Y., Marini F.C., Ayyanan A., Wang R.-Y., Brisken C., Guerra R., Andreeff M. (2010). Epithelial-Mesenchymal Transition-Derived Cells Exhibit Multilineage Differentiation Potential Similar to Mesenchymal Stem Cells. Stem Cells Dayt. Ohio.

[29] Mani S.A., Guo W., Liao M.-J., Eaton E.N., Ayyanan A., Zhou A.Y., Brooks M., Reinhard F., Zhang C.C., Shipitsin M. (2008). The Epithelial-Mesenchymal Transition Generates Cells with Properties of Stem Cells. Cell.

[30] Diehn M., Cho R.W., Lobo N.A., Kalisky T., Dorie M.J., Kulp A.N., Qian D., Lam J.S., Ailles L.E., Wong M. (2009). Association of Reactive Oxygen Species Levels and Radioresistance in Cancer Stem Cells. Nature.

[31] Li X., Lewis M.T., Huang J., Gutierrez C., Osborne C.K., Wu M.-F., Hilsenbeck S.G., Pavlick A., Zhang X., Chamness G.C. (2008). Intrinsic Resistance of Tumorigenic Breast Cancer Cells to Chemotherapy. J. Natl. Cancer Inst..

[32] Jolly M.K., Huang B., Lu M., Mani S.A., Levine H., Ben-Jacob E. (2014). Towards Elucidating the Connection between Epithelial-Mesenchymal Transitions and Stemness. J. R. Soc. Interface.

[33] Yang W.-H., Lan H.-Y., Huang C.-H., Tai S.-K., Tzeng C.-H., Kao S.-Y., Wu K.-J., Hung M.-C., Yang M.-H. (2012). RAC1 Activation Mediates Twist1-Induced Cancer Cell Migration. Nat. Cell Biol..

[34] Wellner U., Schubert J., Burk U.C., Schmalhofer O., Zhu F., Sonntag A., Waldvogel B., Vannier C., Darling D., zur Hausen A. (2009). The EMT-Activator ZEB1 Promotes Tumorigenicity by Repressing Stemness-Inhibiting MicroRNAs. Nat. Cell Biol..

[35] Morel A.-P., Lièvre M., Thomas C., Hinkal G., Ansieau S., Puisieux A. (2008). Generation of Breast Cancer Stem Cells through Epithelial-Mesenchymal Transition. PLoS ONE.

[36] Bhat-Nakshatri P., Appaiah H., Ballas C., Pick-Franke P., Goulet R., Badve S., Srour E.F., Nakshatri H. (2010). SLUG/SNAI2 and Tumor Necrosis Factor Generate Breast Cells with CD44+/CD24- Phenotype. BMC Cancer.

[37] Kröger C., Afeyan A., Mraz J., Eaton E.N., Reinhardt F., Khodor Y.L., Thiru P., Bierie B., Ye X., Burge C.B. (2019). Acquisition of a Hybrid E/M State Is Essential for Tumorigenicity of Basal Breast Cancer Cells. Proc. Natl. Acad. Sci. USA.

[38] Subbalakshmi A.R., Sahoo S., Biswas K., Jolly M.K. (2021). A Computational Systems Biology Approach Identifies SLUG as a Mediator of Partial Epithelial-Mesenchymal Transition (EMT). Cells Tissues Organs.

[39] Siemens H., Jackstadt R., Hünten S., Kaller M., Menssen A., Götz U., Hermeking H. (2011). MiR-34 and SNAIL Form a Double-Negative Feedback Loop to Regulate Epithelial-Mesenchymal Transitions. Cell Cycle Georget. Tex.

[40] Gingold J.A., Fidalgo M., Guallar D., Lau Z., Sun Z., Zhou H., Faiola F., Huang X., Lee D.-F., Waghray A. (2014). A Genome-Wide RNAi Screen Identifies Opposing Functions of Snai1 and Snai2 on the Nanog Dependency in Reprogramming. Mol. Cell.

[41] Lu Z.-Y., Dong R., Li D., Li W.-B., Xu F.-Q., Geng Y., Zhang Y.-S. (2012). SNAI1 Overexpression Induces Stemness and Promotes Ovarian Cancer Cell Invasion and Metastasis. Oncol. Rep..

[42] Wittrup A., Lieberman J. (2015). Knocking down Disease: A Progress Report on SiRNA Therapeutics. Nat. Rev. Genet..

[43] Finlay J., Roberts C.M., Dong J., Zink J.I., Tamanoi F., Glackin C.A. (2015). Mesoporous Silica Nanoparticle Delivery of Chemically Modified SiRNA against TWIST1 Leads to Reduced Tumor Burden. Nanomed. Nanotechnol. Biol. Med..

[44] Lu J., Liong M., Zink J.I., Tamanoi F. (2007). Mesoporous Silica Nanoparticles as a Delivery System for Hydrophobic Anticancer Drugs. Small Weinh. Bergstr. Ger..

[45] Shahin S.A., Wang R., Simargi S.I., Contreras A., Parra Echavarria L., Qu L., Wen W., Dellinger T., Unternaehrer J., Tamanoi F. (2018). Hyaluronic Acid Conjugated Nanoparticle Delivery of SiRNA against TWIST Reduces Tumor Burden and Enhances Sensitivity to Cisplatin in Ovarian Cancer. Nanomed. Nanotechnol. Biol. Med..

[46] Hom C., Lu J., Liong M., Luo H., Li Z., Zink J.I., Tamanoi F. (2010). Mesoporous Silica Nanoparticles Facilitate Delivery of SiRNA to Shutdown Signaling Pathways in Mammalian Cells. Small Weinh. Bergstr. Ger..

[47] Zhang S., Balch C., Chan M.W., Lai H.-C., Matei D., Schilder J.M., Yan P.S., Huang T.H.-M., Nephew K.P. (2008). Identification and Characterization of Ovarian Cancer-Initiating Cells from Primary Human Tumors. Cancer Res..

[48] Liu X., Ory V., Chapman S., Yuan H., Albanese C., Kallakury B., Timofeeva O.A., Nealon C., Dakic A., Simic V. (2012). ROCK Inhibitor and Feeder Cells Induce the Conditional Reprogramming of Epithelial Cells. Am. J. Pathol..

[49] Meng H., Xue M., Xia T., Ji Z., Tarn D.Y., Zink J.I., Nel A.E. (2011). Use of Size and a Copolymer Design Feature to Improve the Biodistribution and the Enhanced Permeability and Retention Effect of Doxorubicin-Loaded Mesoporous Silica Nanoparticles in a Murine Xenograft Tumor Model. ACS Nano.

[50] Wang Z., Lin S., Li J.J., Xu Z., Yao H., Zhu X., Xie D., Shen Z., Sze J., Li K. (2011). MYC Protein Inhibits Transcription of the MicroRNA Cluster MC-Let-7a-1~let-7d via Noncanonical E-Box. J. Biol. Chem..

[51] Wang D.J., Legesse-Miller A., Johnson E.L., Coller H.A. (2012). Regulation of the Let-7a-3 Promoter by NF-ΚB. PLoS ONE.

[52] Careccia S., Mainardi S., Pelosi A., Gurtner A., Diverio D., Riccioni R., Testa U., Pelosi E., Piaggio G., Sacchi A. (2009). A Restricted Signature of MiRNAs Distinguishes APL Blasts from Normal Promyelocytes. Oncogene.

[53] Pelosi A., Careccia S., Sagrestani G., Nanni S., Manni I., Schinzari V., Martens J.H.A., Farsetti A., Stunnenberg H.G., Gentileschi M.P. (2014). Dual Promoter Usage as Regulatory Mechanism of Let-7c Expression in Leukemic and Solid Tumors. Mol. Cancer Res. MCR.

[54] O’Hara S.P., Splinter P.L., Gajdos G.B., Trussoni C.E., Fernandez-Zapico M.E., Chen X.-M., LaRusso N.F. (2010). NFkappaB P50-CCAAT/Enhancer-Binding Protein Beta (C/EBPbeta)-Mediated Transcriptional Repression of MicroRNA Let-7i Following Microbial Infection. J. Biol. Chem..

[55] Brown C.E., Starr R., Martinez C., Aguilar B., D’Apuzzo M., Todorov I., Shih C.-C., Badie B., Hudecek M., Riddell S.R. (2009). Recognition and Killing of Brain Tumor Stem-like Initiating Cells by CD8+ Cytolytic T Cells. Cancer Res..

[56] Roberts C.M., Shahin S.A., Wen W., Finlay J.B., Dong J., Wang R., Dellinger T.H., Zink J.I., Tamanoi F., Glackin C.A. (2017). Nanoparticle Delivery of SiRNA against TWIST to Reduce Drug Resistance and Tumor Growth in Ovarian Cancer Models. Nanomed. Nanotechnol. Biol. Med..

[57] Elliott R.L., Blobe G.C. (2005). Role of Transforming Growth Factor Beta in Human Cancer. J. Clin. Oncol. Off. J. Am. Soc. Clin. Oncol..

[58] Al Moustafa A.-E., Achkhar A., Yasmeen A. (2012). EGF-Receptor Signaling and Epithelial-Mesenchymal Transition in Human Carcinomas. Front. Biosci. Sch. Ed..

[59] Hojo N., Huisken A.L., Wang H., Chirshev E., Kim N.S., Nguyen S.M., Campos H., Glackin C.A., Ioffe Y.J., Unternaehrer J.J. (2018). Snail Knockdown Reverses Stemness and Inhibits Tumour Growth in Ovarian Cancer. Sci. Rep..

[60] Yu F., Yao H., Zhu P., Zhang X., Pan Q., Gong C., Huang Y., Hu X., Su F., Lieberman J. (2007). Let-7 Regulates Self Renewal and Tumorigenicity of Breast Cancer Cells. Cell.

[61] Hahn S., Jackstadt R., Siemens H., Hünten S., Hermeking H. (2013). SNAIL and MiR-34a Feed-Forward Regulation of ZNF281/ZBP99 Promotes Epithelial-Mesenchymal Transition. EMBO J..

[62] Li C.-F., Chen J.-Y., Ho Y.-H., Hsu W.-H., Wu L.-C., Lan H.-Y., Hsu D.S.-S., Tai S.-K., Chang Y.-C., Yang M.-H. (2019). Snail-Induced Claudin-11 Prompts Collective Migration for Tumour Progression. Nat. Cell Biol..

[63] Liu Y., Li H., Feng J., Cui X., Huang W., Li Y., Su F., Liu Q., Zhu J., Lv X. (2013). Lin28 Induces Epithelial-to-Mesenchymal Transition and Stemness via Downregulation of Let-7a in Breast Cancer Cells. PLoS ONE.

[64] Kurrey N.K., Jalgaonkar S.P., Joglekar A.V., Ghanate A.D., Chaskar P.D., Doiphode R.Y., Bapat S.A. (2009). Snail and Slug Mediate Radioresistance and Chemoresistance by Antagonizing P53-Mediated Apoptosis and Acquiring a Stem-like Phenotype in Ovarian Cancer Cells. Stem Cells Dayt. Ohio.

[65] Wang Y.-Y., Yang Y.-X., Zhao R., Pan S.-T., Zhe H., He Z.-X., Duan W., Zhang X., Yang T., Qiu J.-X. (2015). Bardoxolone Methyl Induces Apoptosis and Autophagy and Inhibits Epithelial-to-Mesenchymal Transition and Stemness in Esophageal Squamous Cancer Cells. Drug Des. Devel. Ther..

[66] Liong M., Lu J., Kovochich M., Xia T., Ruehm S.G., Nel A.E., Tamanoi F., Zink J.I. (2008). Multifunctional Inorganic Nanoparticles for Imaging, Targeting, and Drug Delivery. ACS Nano.

[67] Marson A., Levine S.S., Cole M.F., Frampton G.M., Brambrink T., Johnstone S., Guenther M.G., Johnston W.K., Wernig M., Newman J. (2008). Connecting MicroRNA Genes to the Core Transcriptional Regulatory Circuitry of Embryonic Stem Cells. Cell.

[68] Oshima M., Hasegawa N., Mochizuki-Kashio M., Muto T., Miyagi S., Koide S., Yabata S., Wendt G.R., Saraya A., Wang C. (2016). Ezh2 Regulates the Lin28/Let-7 Pathway to Restrict Activation of Fetal Gene Signature in Adult Hematopoietic Stem Cells. Exp. Hematol..

