# Supplementary material for: The Epithelial–Mesenchymal Transcription Factor SNAI1 Represses Transcription of the Tumor Suppressor miRNA let-7 in Cancer
Source: Cancers (Basel). 2021 Mar 23;13(6):1469. doi: 10.3390/cancers13061469 (PMC8004805; doi:10.3390/cancers13061469)
Supplement: Supplementary file 1 [file cancers-13-01469-s001.pdf]

cancers

MDPI

Supplementary Material: The Epithelial–Mesenchymal Transcription Factor *SNAI1* Represses Transcription of the Tumor Suppressor miRNA *let-7* in Cancer

Wang H, Chirshev E, Hojo N , Suzuki T, Bertucci A, Pierce M, Perry C, Wang R, Zink J, Glackin CA, Ioffe YJ and Unternaehrer JJ

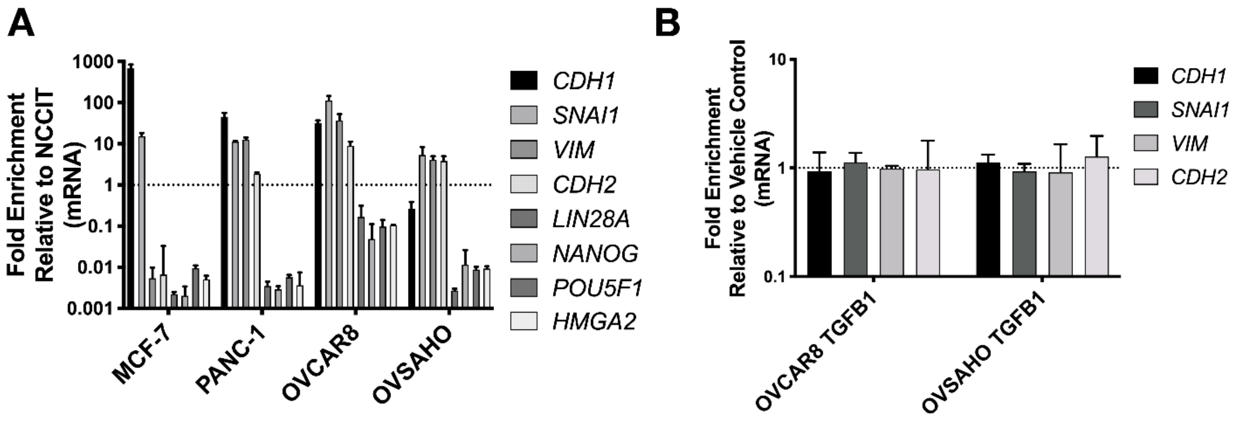

Figure S1. Parental cell line gene expression comparison; EMT is not induced by TGFB1 treatment in ovarian cancer cell lines. (**A**) EMT status and stemness gene expression level comparison in parental cell lines. mRNA expression levels of epithelial (*CDH1*), of mesenchymal (*SNAI1*, *VIM* and *CDH2*), and of stemness markers (*LIN28A*, *NANOG*, *POU5F1*, and *HMGA2*, left to right) were analyzed using RT-qPCR. The expression levels were normalized to levels in NCCIT embryonal carcinoma cells (positive control for pluripotency gene expression), indicated as the dotted line. (**B**) TGFB1 does not induce EMT in OVSAHO or OVCAR8. mRNA expression levels of epithelial (*CDH1*) and mesenchymal (*SNAI1, VIM and CDH2*) factors (from left to right) were analyzed using RT-qPCR after OVCAR8 (left) and OVSAHO (right) were treated with TGFB1. The expression levels of markers in control groups (cells treated with vehicle control) were normalized to 1, indicated as the dotted line.

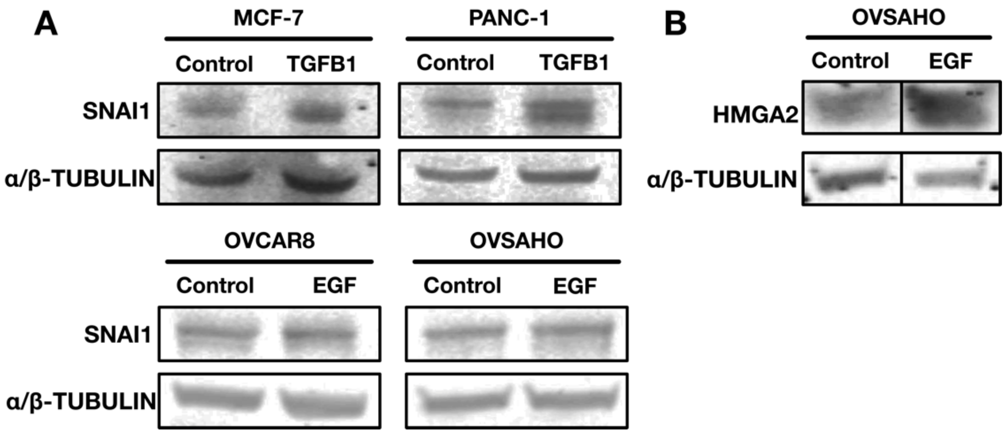

Figure S2. Western blot data for growth factor treatment. (**A**) Western blot analysis for protein expression of SNAI1 in MCF-7, PANC-1, OVCAR8, and OVSAHO (samples from Fig. 1C). α/β-TUBULIN was used as a loading control. (**B**) Western blot analysis for protein expression of HMGA2 in OVSAHO (samples from Fig. 1D). α/β-TUBULIN was used as a loading control.

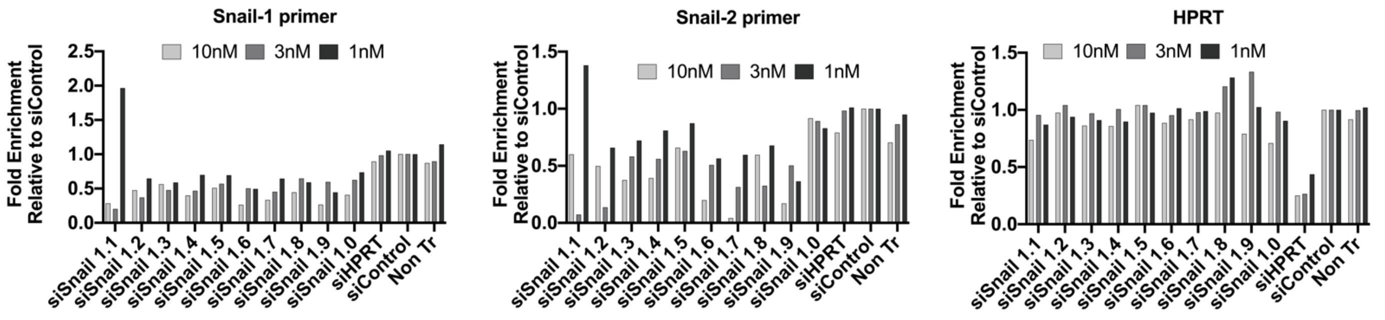

Figure S3. The effectiveness of different DsiRNAs. Twelve DsiRNAs were screened for their effectiveness at SNAI1 knockdown in HEK293T cells. Lipofectamine RNAiMax (Life Technologies) was used for transfection of 1, 3, or 10nM DsiRNAs. After 24h, levels of *SNAI1* RNA using a 5′ (*SNAI1*-1) and a 3′ (*SNAI1*-2) primer pair were determined with RT-qPCR. As a positive control, HPRT was targeted; siControl was used as a negative control. siSnail 1.7 was chosen as most effective in dose response for SNAI1 knockdown. siSnail 1.6 were also used for *in vitro* knockdown with similar results.

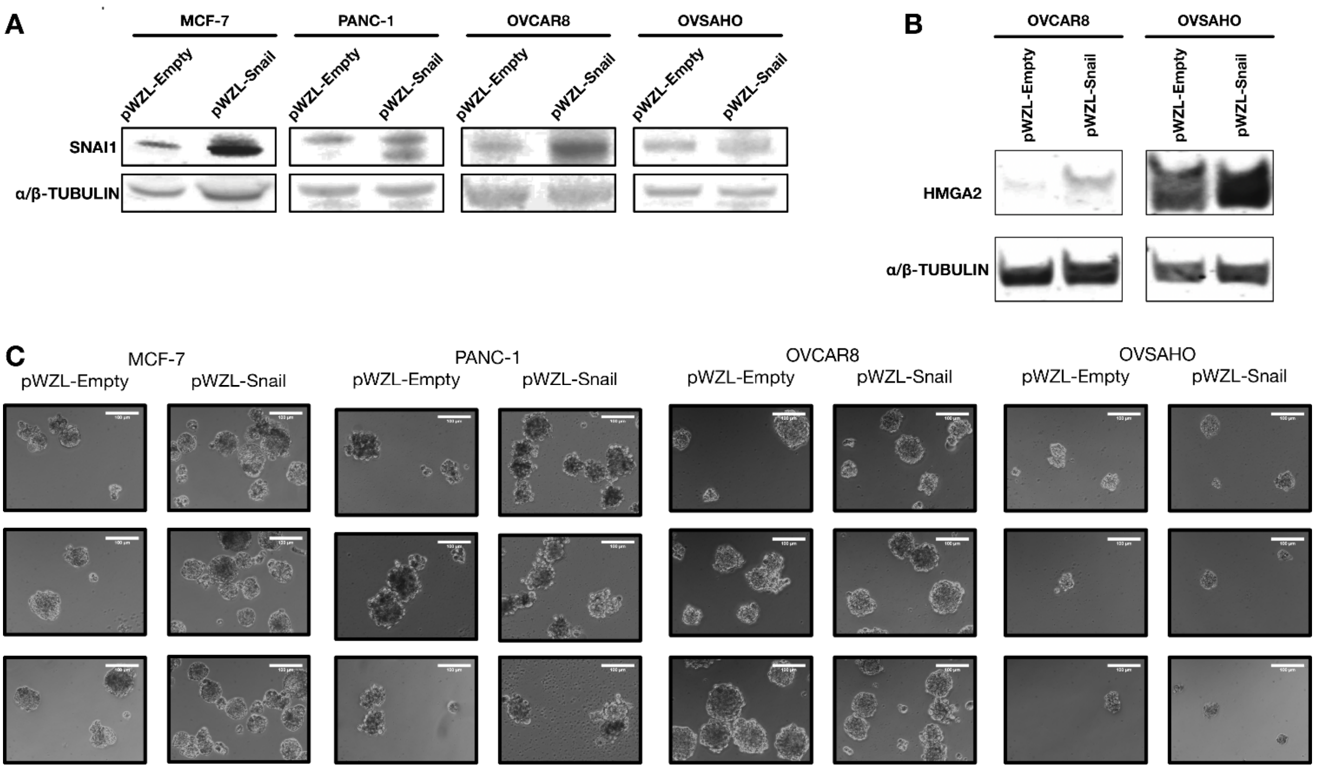

Figure S4. Western blot data and pictures of spheroids formed with SNAI1 overexpression**.** (**A**) Western blot analysis for protein expression of SNAI1 in MCF-7, PANC-1, OVCAR8, and OVSAHO (samples from Fig. 2C). α/β-TUBULIN was used as a loading control. (**B**) Western blot analysis for protein expression of HMGA2 in OVCAR8 and OVSAHO (samples from Fig. 2D). α/β-TUBULIN was used as a loading control. (**C**) Phase contrast images (3 representative images) of spheroids formed from MCF-7, PANC-1, OVCAR8 and OVSAHO are presented; in each panel, the spheroids formed from control group (cells transduced with pWZL-Empty) are presented on the left, those from pWZL-Snail group are on the right (quantified in Fig. 2E). Scale bar = 100 μm.

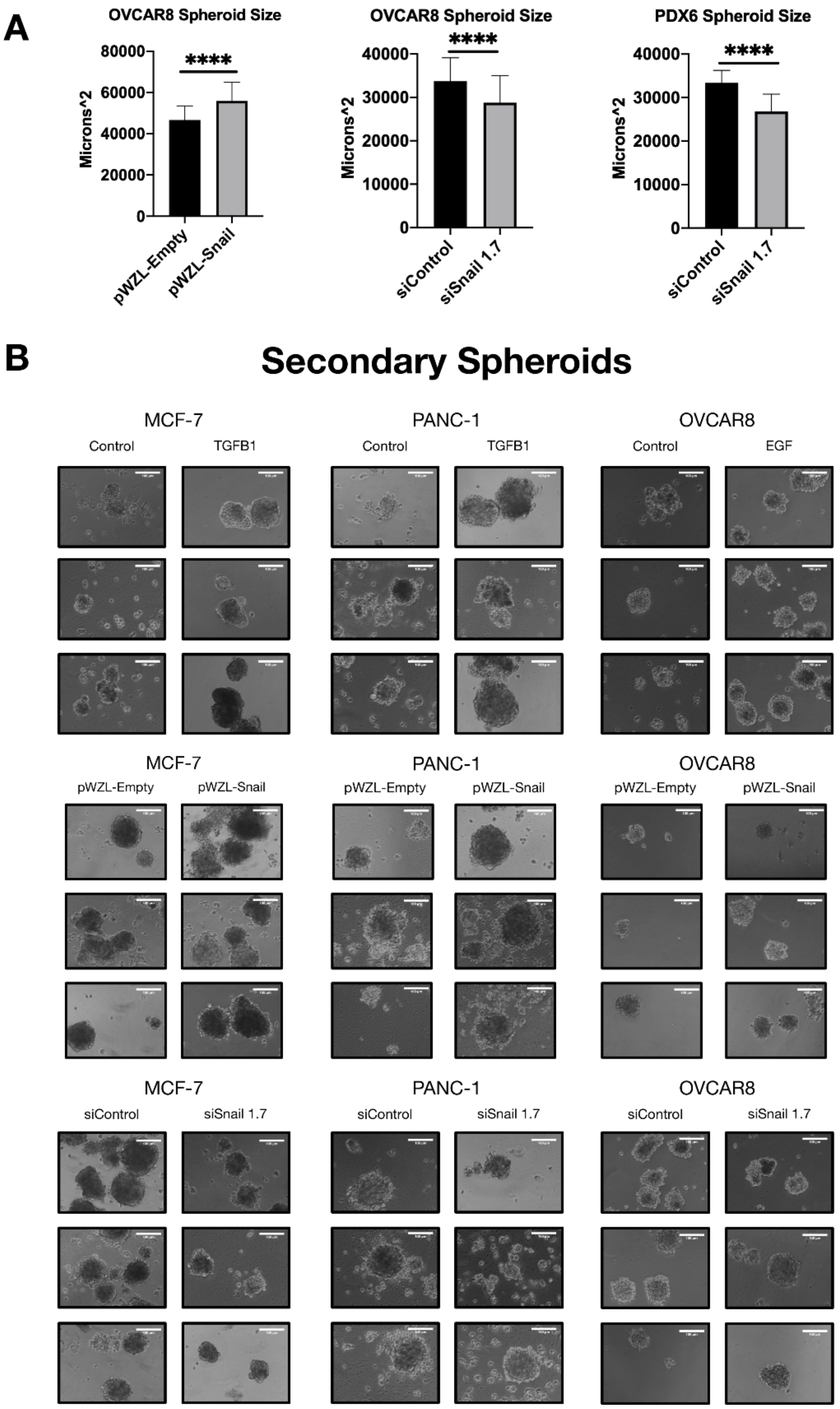

Figure S5. Higher level of *SNAI1* is associated with larger size of spheroids and higher number of secondary spheroids formed. (**A**) The quantification of spheroid size (unit: microns^2) for OVCAR8 in *SNAI1* overexpression (left), SNAI1 knockdown (middle) and PDX6 cells in *SNAI1* knockdown in vitro (right); (**B**) Phase contrast images of spheroids (Second passage) for EMT induction via TGFB1/EGF (top panel), *SNAI1* retroviral overexpression (middle panel) and *SNAI1* knockdown (lower panel). In each panel, pictures of spheroids of MCF-7, PANC-1, OVCAR8 are shown (left to right). Scale bar = 100 μm.

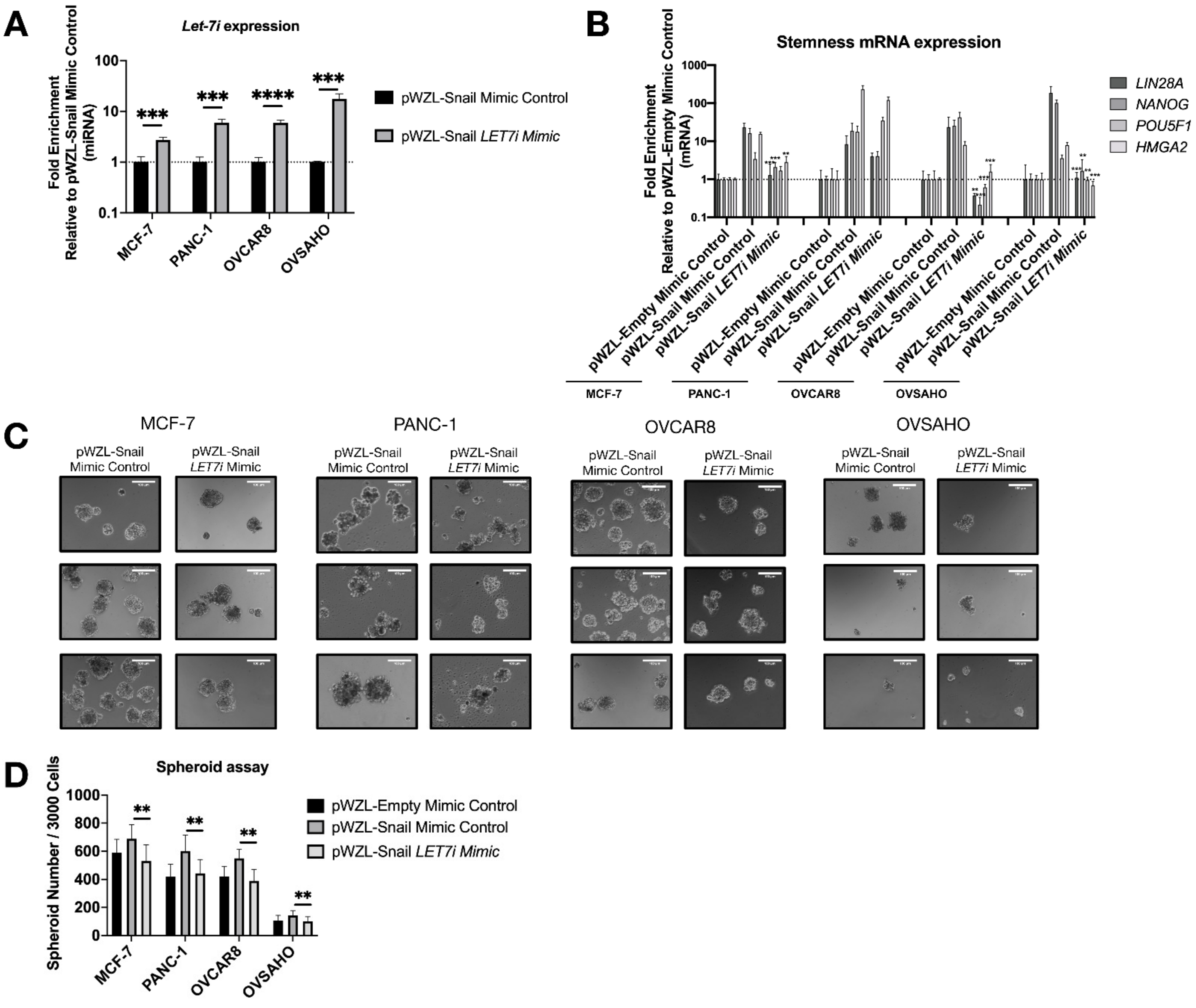

Figure S6. *Let-7i* overexpression rescues the increase in stemness resulting from *SNAI1* overexpression. (**A**) For the rescue experiment, cells transduced with pWZL-Snail were also transfected with control mimic or *let-7i* mimic afterwards; miRNA expression levels of *let-7i* were analyzed using RT-qPCR. The expression levels of *let-7i* in control groups (cells transduced with pWZL-Snail then transfected with Mimic Control) were normalized to 1. (**B**) RT-qPCR analysis for stemness markers (*LIN28A, NANOG, POU5F1,* and *HMGA2*) in MCF-7, PANC-1, OVCAR8 and OVSAHO are presented, note only the cells transduced with pWZL-Empty then transfected with Mimic Control were normalized to 1. (**C**) Phase contrast images of spheroids for MCF-7, PANC-1, OVCAR8 and OVSAHO (left to right). In each panel, pictures from control mimic group are shown on the left, those from *let-7i* mimic group on the right. Scale bar = 100 µm. (**D**) The quantification of number of spheroids per 3000 cells formed from MCF-7, PANC-1, OVCAR8 and OVSAHO in the rescue experiment.

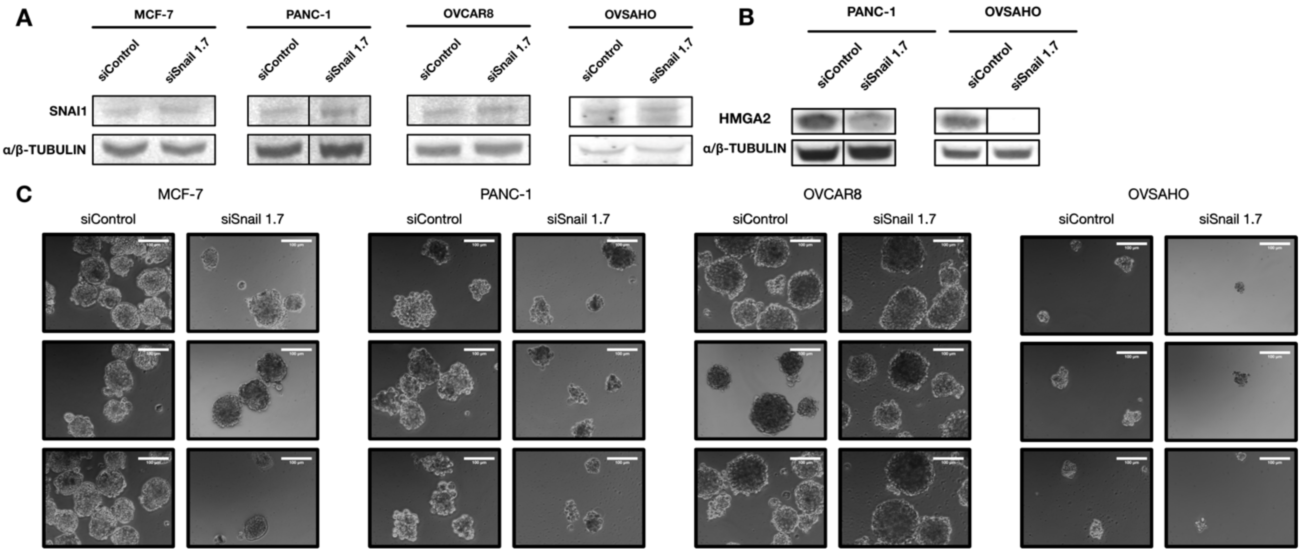

Figure S7. Western blot data and pictures of spheroids formed in SNAI1 knockdown in cancer cell lines. (**A**) Western blot analysis for protein expression of SNAI1 in MCF-7, PANC-1, OVCAR8, and OVSAHO (quantified in Fig. 3C). α/β-TUBULIN was used as a loading control. (**B**) Western blot analysis for protein expression of HMGA2 in PANC-1 and OVSAHO (quantified in Fig. 3D). α/β-TUBULIN was used as a loading control. Samples were harvested after 24 hours (MCF-7, OVCAR8 and OVSAHO) or 72 hours (PANC-1). (**C**) Phase contrast images (3 representative images) of spheroids formed from MCF-7, PANC-1, OVCAR8 and OVSAHO are presented. In each panel, the spheroids formed from control group (cells transduced with siControl) are presented on the left, those from siSnail 1.7 group are on the right (quantified in Fig. 3E). Scale bar = 100 μm.

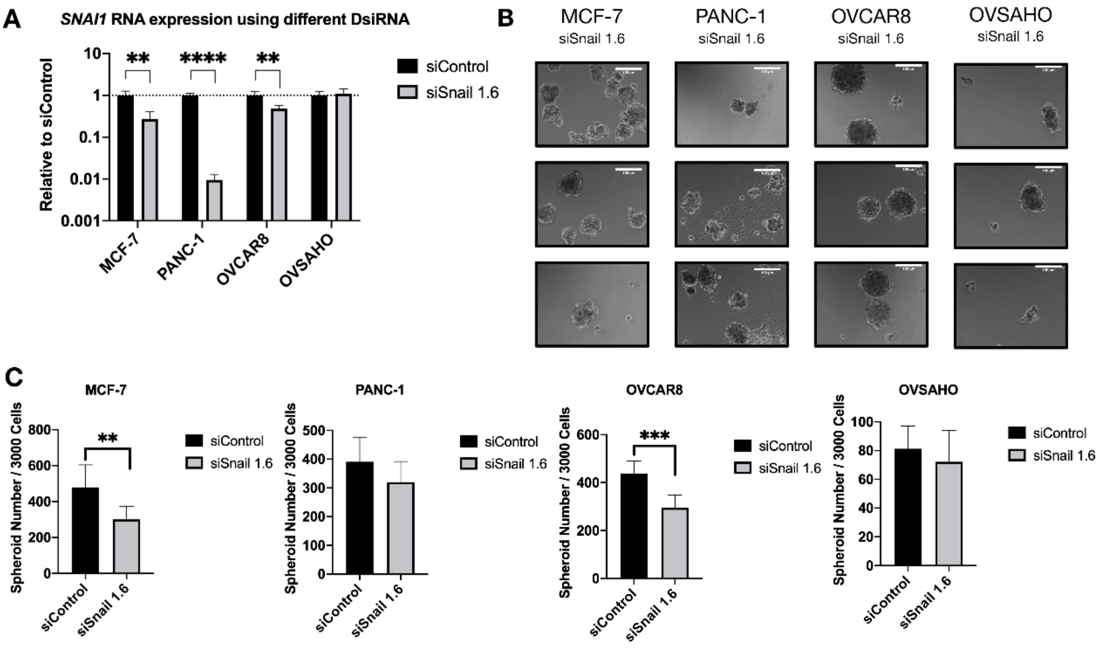

Figure S8. Knockdown of *SNAI1* with a second DsiRNA. (**A**) mRNA expression level of *SNAI1* in MCF-7, PANC-1, OVCAR8 and OVSAHO after treatment of DsiRNA 1.6 was analyzed using RT-qPCR. The expression level of *SNAI1* in control groups (cells treated with siControl) was normalized to 1, indicated as the dotted line. (**B**) Phase contrast images of spheroids formed from siSnail 1.6 group. Scale bar = 100 μm. (**C**) The quantification of number of spheroids formed per 3000 cells.

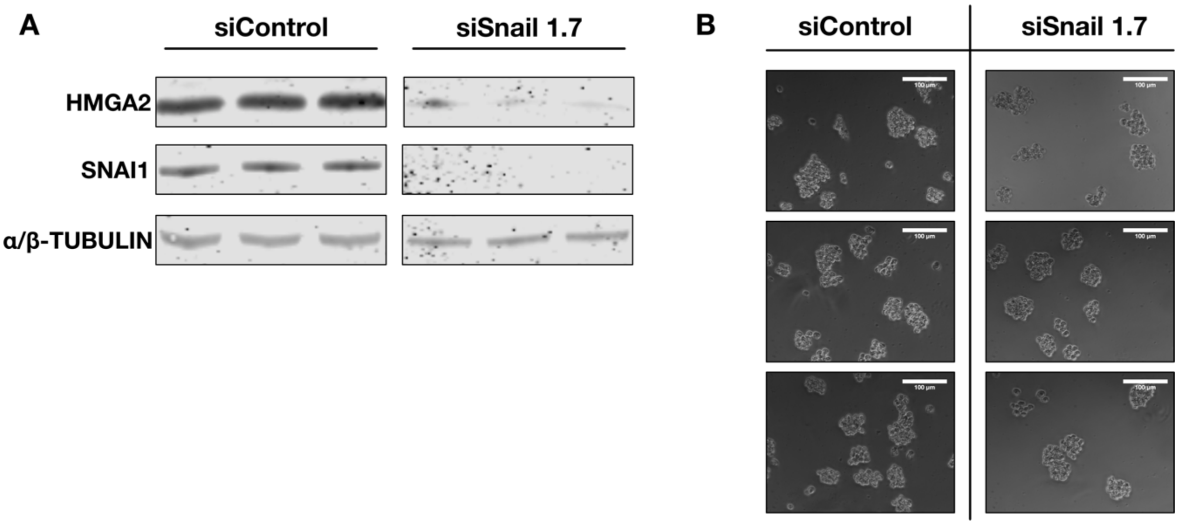

Figure S9. Western blot data and pictures of spheroids formed in SNAI1 knockdown in PDX cells in vitro. (**A**) Western blot analysis for protein expression of SNAI1 and HMGA2 in PDX6 in vitro (quantified in Fig. 4C and D). α/β-TUBULIN was used as a loading control. Triplicates of each group are presented. (**B**) Phase contrast images (3 representative images) of spheroids formed from PDX6 are presented (quantified in Fig. 4E). Scale bar = 100 µm.

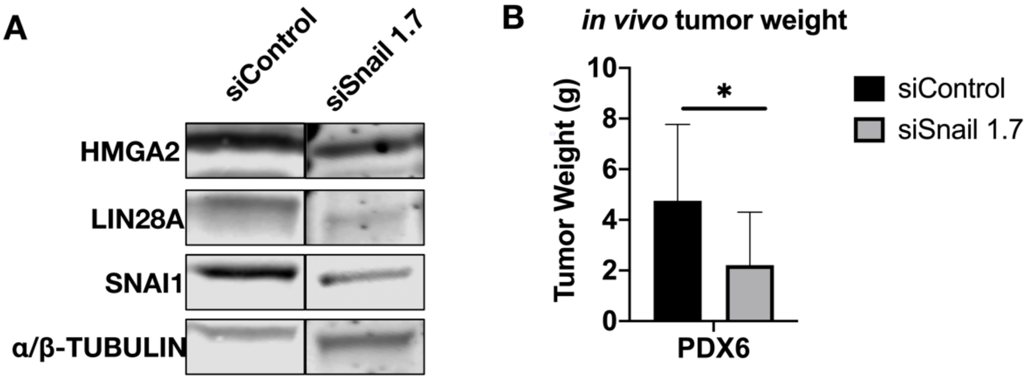

Figure S10. Western blot data and tumor weight comparison for *SNAI1* knockdown in PDX cells in vivo. (**A)** Western blot analysis for protein expression of SNAI1 HMGA2 and LIN28A in PDX6 *in vivo* (quantified in Fig. 5C and D). α/β-TUBULIN was used as a loading control. (**B**) The average tumor weights in mice treated with siControl vs. siSnail in vivo.

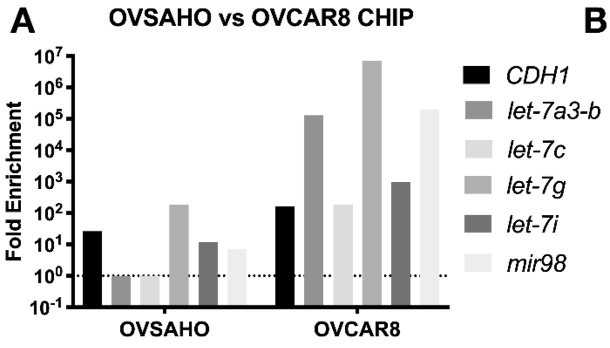

B

	CDH1	*let-7a3-b*	*let-7c*	*let-7g*	*let-7i*	*mir98*
Control	6.05732488	0.03138349	0.00449943	0.00025097	0.60049700	0.0000011922
TGFB1	358.25656400	3.09103324	1.00000000	1.00000000	403.67517800	523.7606820000

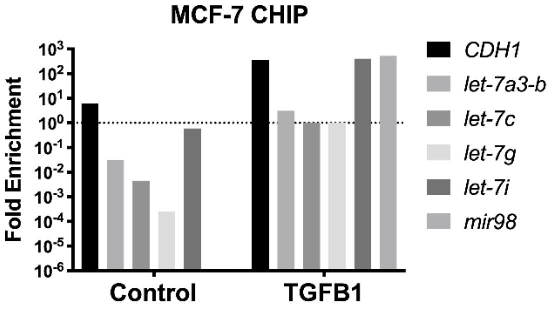

Figure S11. SNAI1 binds to *let-7* promoters demonstrated by ChIP. Chromatin immunoprecipitation (ChIP) analysis of SNAI1 binding to *let-7* promoter regions. SNAI1 binding *to let-7a3-b, let-7c, let-7g, let-7i, mir98* was measured as fold enrichment relative to IgG negative control. *CDH1* was used as an internal positive control. The ChIP data are representative of two experiments. (**A**) Parental cell lines of OVCAR8 and OVSAHO were collected and their levels of SNAI1 binding to *let-7* promoter were measured by ChIP. (**B**) MCF-7 cells were treated with TGFB1. Shown are cells treated with vehicle control vs TGFB1. The table above shows the exact fold enrichment for each primer.

Western Blot whole membrane used in manuscript.

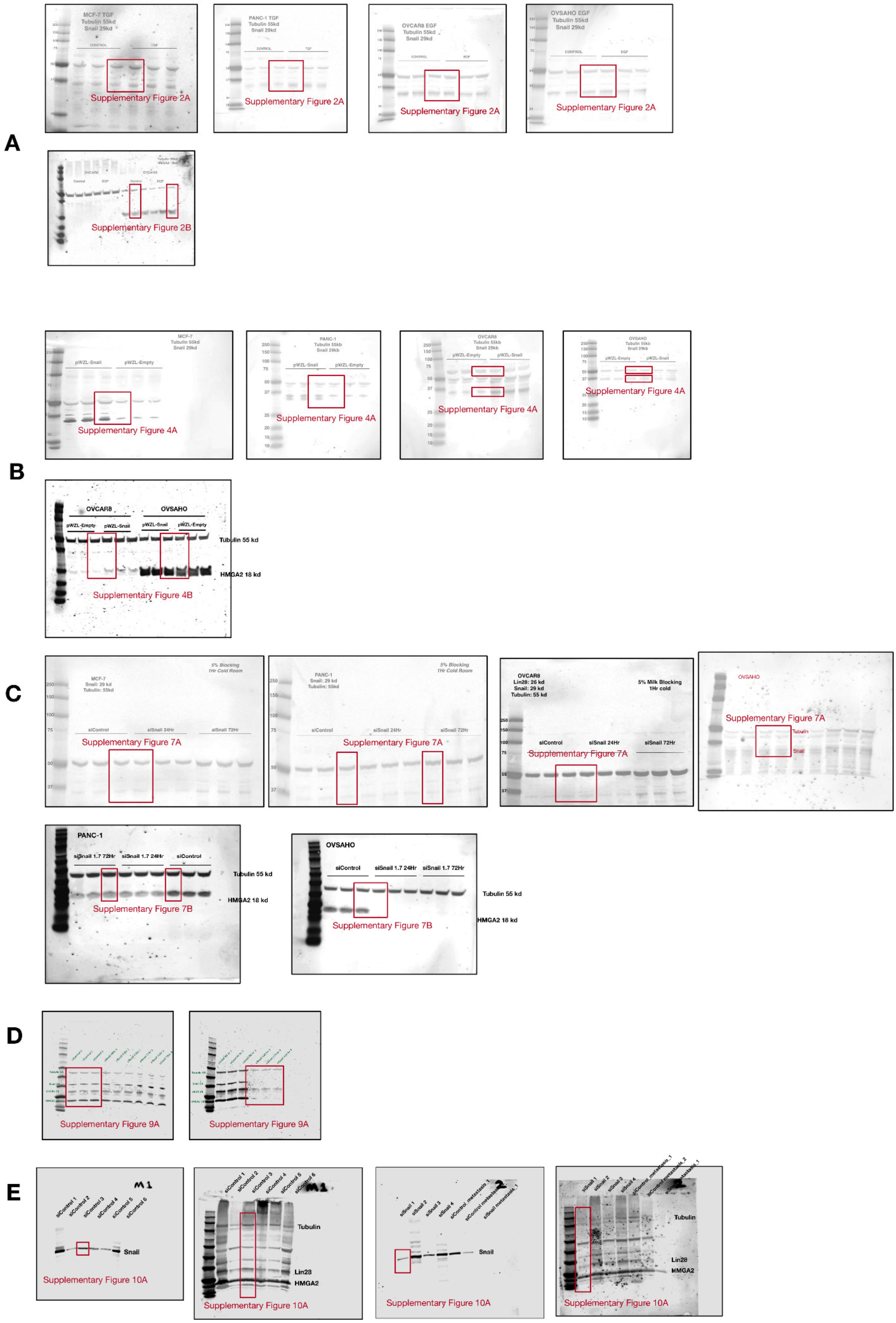

Figure S12. Uncropped Western blot membranes used in manuscript. (**A**) Refers to Supplementary Figure 2. The Western blot results for SNAI1 in TGFB1/EGF treatments for MCF-7, PANC-1, OVCAR8 and OVSAHO are presented here from left to right (one cell line per blot). In each blot, the left three lanes were samples from control group, the right three lanes were samples from treatment group (upper panel). The blot for HMGA2 bands in OVCAR8 and OVSAHO after EGF treatment is presented in lower panel. The lanes from control and EGF group of OVCAR8 and OVSAHO are indicated in the blot. One lane from each group was picked and presented in the figure. The position of TUBULIN bands, HMGA2 bands and SNAI1 bands could be determined based on the molecular weight markers. Additionally, the signal for TUBULIN, HMGA2, LIN28 is visible in the 700 nm channel, while that for SNAI1 is visible in the 800 nm channel; this also applies to B, C, D and E. (**B**) Refers to Supplementary Figure 4. The Western blot results for SNAI1 in *SNAI1* overexpression samples for MCF-7, PANC-1, OVCAR8 and OVSAHO are presented from left to right (one cell line per blot). The result for HMGA2 in OVCAR8 and OVSAHO is presented in the lower panel. The lanes from pWZL-Empty and pWZL-Snail group are indicated in each blot. One lane from each group was picked and presented in the figure. The position of TUBULIN bands and SNAI1 bands could be determined based on the molecular weight markers. (**C**) Refers to Supplementary Figure 7. The Western blot results for SNAI1 in *SNAI1* knockdown samples for MCF-7, PANC-1, OVCAR8 and OVSAHO are presented from left to right (one cell line per blot). The Western blot results for HMGA2 in *SNAI1* knockdown samples for PANC-1 and OVSAHO are presented (one cell line per blot). The lanes from siControl; siSnail 1.7 24Hr and siSnail 1.7 48 Hr group are indicated in each blot. One lane from each group (siControl and siSnail 1.7 24Hr) was picked and presented in the figure. The position of TUBULIN bands and HMGA2 bands are indicated on the blot. (**D**) Refers to Figure 4. The Western blot results of PDX6 cells treated with siSnail 1.7 *in vitro*. From left to right were siControl samples, siSnail 1.7 48 hrs, siSnail 1.7 72 hrs (left blot), siSnail 1.7 96 hrs, siSnail 120 hrs (right blot), each in triplicate. The samples from siControl and siSnail 120 hrs were picked and presented in the figure. The position of TUBULIN bands, SNAI1 bands, LIN28 bands and HMGA2 bands are noted on the blots. (**E**) Refers to Figure 5. The Western blot results of PDX6 cells treated with siSnail 1.7 in vivo. The two blots on the left are identical blots scanned at 800 nm (left) and 700 nm (right) to visualize SNAI1 (left) and TUBULIN, LIN28A, HMGA2 (right). The same applies to the two blots on the right. In the two blots on the left, each lane represented a primary tumor sample from one mouse from siControl group. In the two blots on the right, the first four lanes each represented a primary tumor sample from one mouse from siSnail group. The last three lanes are metastasis tumor samples that are from siControl and siSnail (not presented in manuscript). The position of TUBULIN, SNAI1, LIN28A, and HMGA2 bands are noted on the blots.

Table S1. *Let-7* promoter region length, locations and E-box (CANNTG) locations.

***let-7* Member**	**E-box Starting Site: bp Ppstream of TSS**	**Promoter Region Starting Site to Ending Site: bp Upstream of TSS**
let-7a1-f1[1]	-8924, -8431	-9775 to -8286
let-7a3-b[2]	-1688, -1581, -1302, -1234	-1960 to -917
let-7c[3]	-1783, -1526, -1501, -1326, -1279, -640, -481, -247, -197	-1804 to 0
let-7g[4]	-11259, -9824, -9194, -9045	-11397 to -9000
let-7i[5]	-1545, -891, -375	-2303 to -39
mir98[6]	-4479, -2749, -2217, -1249	-5032 to - 536

Table S2. Sequences of primers used in RT-qPCR.

**primers**	**Forward**	**Reverse**
ACTB	5' - TGAAGTGTGACGTGGACATC - 3'	5' - GGAGGAGCAATGATCTTGAT - 3'
SNAI1 Snail-1	5' - CACTATGCCGCGCTCTTTC - 3'	5' - GGTCGTAGGGCTGCTGGAA - 3'
SNAI1 Snail-2	5′ - GGCTGCTACAAGGCCAT - 3′	5′ - GCACTGGTACTTCTTGACATCT - 3′
VIM	5′ - GAGTCCACTGAGTACCGGAGAC - 3′	5′ - TGTAGGTGGCAATCTCAATGTC - 3′
CDH1	5′ - TGCCCAGAAAATGAAAAAGG - 3′	5′ - GTGTATGTGGCAATGCGTTC - 3′
CDH2	5′ - GAGGAGTCAGTGAAGGAGTCA - 3′	5′ - GGGAAGTTGATTGGAGGGATG - 3′
NANOG	5' - CAAAGGCAAACAACCCACTT - 3'	5' - TCTGCTGGAGGCTGAGGTAT - 3'
LIN28A	5' - GAGCATGCAGAAGCGCAGATCAAA - 3'	5' - TATGGCTGATGCTCTGGCAGAAGT - 3'
POU5F1	5' - AAGCGATCAAGCAGCGACTAT - 3'	5' - GGAAAGGGACCGAGGAGTACA - 3'
HMGA2	5' - GCAGCCGTCCACTTCAG - 3'	5' - TCTTCCCCTGGGTCTCTTAG - 3'
HPRT	5′ - GGCTTATATCCAACACTTCG GGG - 3′	5′ - GACTTTGCTTTCCTTGGTCAG - 3′

Table S3. Sequences of primers used in chromatin immunoprecipitation.

ChIP primers	Forward	Reverse
Let-7a3-b	5' - GTCATCCTCATGCTACACTCTG - 3'	5' - AAAGGAGGAGGGAGGGAAA - 3'
Let-7c	5' - ACTCAGATGCTCCTCCTAGAC - 3'	5' - CTCCTCCTGATTTCACTTCCTTT - 3'
Let-7g	5' - GGTACTCGATCTGTCCTTGTTG - 3'	5' - GGAAGAGTTGCACTATCCTGTT - 3'
Let-7i	5' - GGAGGGATTTCAGGCAAGAT - 3'	5' - ATGAAGGGACCCAATCCTTC - 3'
Mir98	5' - TGACTGTGTCTGCCGTAATG - 3'	5' - AGTGTGACTGTTGAGTGAGAAG - 3'
CDH1	5′ – GTTTGTTTGTTTTCCAAC – 3′	5′ – GCAAGGCTATGTCTCAAAAG – 3′

Table S4. Sequences of siRNA oligonucleotides.

RNA oligonucleotides	Sense (5′–3′)	Antisense (5′–3′)
siSnail 1.1	rArGrArUrGrUrCrArArGrArArGrUrArCrC rArGrUrGrCrCAG	rCrUrGrGrCrArCrUrGrGrUrArCrUrUrCrUr UrGrArCrArUrCrUrGrA
siSnail 1.2	rUrGrUrGrArCrUrArArCrUrArUrGrCrArA rUrArArUrCrCAC	rGrUrGrGrArUrUrArUrUrGrCrArUrArGrUr UrArGrUrCrArCrArCrC
siSnail 1.3	rUrCrArArCrUrGrCrArArArUrArCrUrGrC rArArCrArArGGA	rUrCrCrUrUrGrUrUrGrCrArGrUrArUrUrUr GrCrArGrUrUrGrArArG
siSnail 1.4	rGrCrGrArGrCrUrGrCrArGrGrArCrUrCrU rArArUrCrCrAGA	rUrCrUrGrGrArUrUrArGrArGrUrCrCrUrGr CrArGrCrUrCrGrCrUrG
siSnail 1.5	rGrGrCrCrUrArGrCrGrArGrUrGrGrUrUrC rUrUrCrUrGrCGC	rGrCrGrCrArGrArArGrArArCrCrArCrUrCr GrCrUrArGrGrCrCrGrU
siSnail 1.6	rCrUrArCrArGrGrArCrArArArGrGrCrUrG rArCrArGrArCTC	rGrArGrUrCrUrGrUrCrArGrCrCrUrUrUrGr UrCrCrUrGrUrArGrCrU
siSnail 1.7	rGrCrCrArArUrGrCrUrCrArUrCrUrGrGrG rArCrUrCrUrGTC	rGrArCrArGrArGrUrCrCrCrArGrArUrGrAr GrCrArUrUrGrGrCrArG
siSnail 1.8	rArCrUrGrUrGrArGrUrArArUrGrGrCrUrG rUrCrArCrUrUGT	rArCrArArGrUrGrArCrArGrCrCrArUrUrAr CrUrCrArCrArGrUrCrC
siSnail 1.9	rCrArArGrArUrGrCrArCrArUrCrCrGrArA rGrCrCrArCrACG	rCrGrUrGrUrGrGrCrUrUrCrGrGrArUrGrUr GrCrArUrCrUrUrGrArG
siSnail 1.0	rUrUrCrCrCrGrGrGrCrArArUrUrUrArArC rArArUrGrUrCTG	rCrArGrArCrArUrUrGrUrUrArArArUrUrGr CrCrCrGrGrGrArArArC
siHPRT	rGrCrCrArGrArCrUrUrUrGrUrUrGrGrArU rUrUrGrArArATT	rArArUrUrUrCrArArArUrCrCrArArCrArAr ArGrUrCrUrGrGrCrUrU
siControl	rCrArUrArUrUrGrCrGrCrGrUrArUrArGrU rCrGrCrGrUrUAG	rCrUrArArCrGrCrGrArCrUrArUrArCrGrCr GrCrArArUrArUrGrGrU
siSnail 1.7 mod	mGmCrCmArAmUrGrCrUrCrAmUrCmUr GmGrGmArCrUrCrUrGmUC	rGrAmCrArGrArGrUmCrCmCrAmGrArUrG rArGrCrArUrUmGrGmCmAmG
siControl mod	mCmArUmArUmUrGrCrGrCrGmUrAmUr AmGrUmCrGrCrGrUrUmAG	rCrUmArArCrGrCrGmArCmUrAmUrArCrG rCrGrCrArArUmArUmGmGmU

Supplementary References:

1. Wang, Z.; Lin, S.; Li, J.J.; Xu, Z.; Yao, H.; Zhu, X.; Xie, D.; Shen, Z.; Sze, J.; Li, K.; et al. MYC Protein Inhibits Transcription of the MicroRNA Cluster MC-Let-7a-1~let-7d via Noncanonical E-Box. *J. Biol. Chem.* **2011**, *286*, 39703–39714, doi:10.1074/jbc.M111.293126.
2. Wang, D.J.; Legesse-Miller, A.; Johnson, E.L.; Coller, H.A. Regulation of the Let-7a-3 Promoter by NF-KB. *PloS One* **2012**, *7*, e31240, doi:10.1371/journal.pone.0031240.
3. Pelosi, A.; Careccia, S.; Sagrestani, G.; Nanni, S.; Manni, I.; Schinzari, V.; Martens, J.H.A.; Farsetti, A.; Stunnenberg, H.G.; Gentileschi, M.P.; et al. Dual Promoter Usage as Regulatory Mechanism of Let-7c Expression in Leukemic and Solid Tumors. *Mol. Cancer Res. MCR* **2014**, *12*, 878–889, doi:10.1158/1541-7786.MCR-13-0410.

4. Chen, K.-C.; Hsieh, I.-C.; Hsi, E.; Wang, Y.-S.; Dai, C.-Y.; Chou, W.-W.; Juo, S.-H.H. Negative Feedback Regulation between MicroRNA Let-7g and the OxLDL Receptor LOX-1. *J. Cell Sci.* **2011**, *124*, 4115–4124, doi:10.1242/jcs.092767.
5. O'Hara, S.P.; Splinter, P.L.; Gajdos, G.B.; Trussoni, C.E.; Fernandez-Zapico, M.E.; Chen, X.-M.; LaRusso, N.F. NFkappaB P50-CCAAT/Enhancer-Binding Protein Beta (C/EBPbeta)-Mediated Transcriptional Repression of MicroRNA Let-7i Following Microbial Infection. *J. Biol. Chem.* **2010**, *285*, 216–225, doi:10.1074/jbc.M109.041640.
6. Yu, R.M.K.; Chaturvedi, G.; Tong, S.K.H.; Nusrin, S.; Giesy, J.P.; Wu, R.S.S.; Kong, R.Y.C. Evidence for MicroRNA-Mediated Regulation of Steroidogenesis by Hypoxia. *Environ. Sci. Technol.* **2015**, *49*, 1138–1147, doi:10.1021/es504676s.
